# Zebrafish as a Model Organism for Post-Traumatic Stress Disorder: Insights into Stress Mechanisms and Behavioral Assays

**DOI:** 10.3390/biology14080939

**Published:** 2025-07-25

**Authors:** Alexey Sarapultsev, Maria Komelkova, Oleg Lookin, Sergey Khatsko, Alexander Zhdanov, Stanislav Fedorov, Evgenii Gusev, Alexander Trofimov, Tursonjan Tokay, Desheng Hu

**Affiliations:** 1 Institute of Immunology and Physiology, Ural Branch of the Russian Academy of Science, 106 Pervomaiskaya Street, 620049 Ekaterinburg, Russia; gusev36@mail.ru; 2 Russian–Chinese Education and Research Center of System Pathology, South Ural State University, 76 Lenin Prospekt, 454080 Chelyabinsk, Russia; mkomelkova@mail.ru (M.K.); fedorovstas2016@yandex.ru (S.F.); 3 National Scientific Medical Center, Astana 010009, Kazakhstan; lookinoleg@gmail.com; 4 Anatomical and Physiological Experimental Laboratory, Department of Experimental Biology and Biotechnology, Institute of Natural Sciences and Mathematics, 48 Kuybysheva Str., 620026 Ekaterinburg, Russia; hardscore@mail.ru (S.K.); sanya.zhdanov.1996@mail.ru (A.Z.); 5 Biology Department, School of Sciences and Humanities, Nazarbayev University, 53 Kabanbai Batyr Ave., Astana 010000, Kazakhstan; alexander.n.trofimov@gmail.com (A.T.); tursonjan.tokay@nu.edu.kz (T.T.); 6 Department of Integrated Traditional Chinese and Western Medicine, Union Hospital, Tongji Medical College, Huazhong University of Science and Technology, Wuhan 430022, China; desheng.hu@hust.edu.cn; 7 Hubei Key Laboratory of Biological Targeted Therapy, China-Russia Medical Research Center for Stress Immunology, Union Hospital, Tongji Medical College, Huazhong University of Science and Technology, Wuhan 430022, China

**Keywords:** zebrafish (*Danio rerio*), post-traumatic stress disorder, experimental models, stress-related neurophysiological alterations, molecular mechanisms, behavioral changes

## Abstract

The zebrafish (*Danio rerio*) has emerged as a powerful model organism for investigating the mechanisms of post-traumatic stress disorder (PTSD), offering unique advantages in translational relevance, genetic trackability, and cost-effectiveness. As a logical continuation of our recent systematic review, this manuscript critically examines the spectrum of experimental strategies used to model PTSD in zebrafish, with a focus on the comparative efficacy and validity of acute, chronic, and complex stress paradigms. Among these, 14–15-day chronic unpredictable stress (CUS/UCS) protocols are identified as the gold standard, reliably inducing core PTSD-like phenotypes—such as anxiety-like behavior, cortisol dysregulation, and neuroinflammatory gene activation. We discuss the influence of environmental, developmental, and genetic factors on stress responses, and highlight the importance of standardized behavioral and molecular endpoints for model validation. While alternative paradigms—including acute, social, pharmacological, and predator-based models—offer mechanistic insights, their translational relevance remains limited without further refinement. We conclude by outlining future directions for zebrafish-based PTSD research, emphasizing the need for protocol harmonization, integration of multi-modal readouts, and exploration of individual variability to enhance the translational value of this model system.

## Simple Summary

Post-traumatic stress disorder is a serious mental health condition that can develop after experiencing or witnessing traumatic events. To better understand how this condition affects the brain and behavior, scientists use animal models that can mimic aspects of trauma and stress. In this study, we review the use of zebrafish—a small tropical freshwater fish—as a model for studying post-traumatic stress. Zebrafish are increasingly used because they share many important biological features with humans, respond to stress in similar ways, and can be studied at all stages of development. We describe how different stress models, such as exposure to predators, social isolation, or unpredictable stressful events, affect zebrafish behavior and biology. Importantly, we also highlight the fish’s ability to learn from experience, remember traumatic events, and show long-term changes in behavior—similar to symptoms seen in people with post-traumatic stress. Our analysis suggests that zebrafish are a valuable and affordable research tool for discovering how stress-related disorders develop and how they might be treated. Understanding stress responses in zebrafish can help researchers develop better ways to prevent and manage trauma-related mental health conditions in humans.

## 1. Introduction

The increasing prevalence of Post-Traumatic Stress Disorder (PTSD) and its significant impact on individuals have spurred extensive research aimed at uncovering its underlying mechanisms and developing effective treatments. Traditionally, rodent models have been the cornerstone of PTSD research due to their physiological and genetic resemblance to humans. However, these models come with certain limitations, such as high costs, ethical concerns, and challenges in genetic manipulation, which have led researchers to seek alternative model organisms. In recent years, zebrafish (*Danio rerio*) have gained recognition as a valuable model for studying PTSD due to their distinct combination of biological, genetic, and behavioral traits [1,2,3,4].

Zebrafish are particularly well-suited for investigating stress-related disorders like PTSD due to several key attributes. They are genetically tractable, reproduce rapidly, and share many physiological and molecular pathways with humans [2,5,6]. Although their nervous system is less complex than that of humans, the genetic makeup of zebrafish is well documented, with a nucleotide sequence that closely resembles other vertebrates [7]. The complete sequencing of the zebrafish genome has revealed an 87% similarity to the human genome, with approximately 70% of their genes having human counterparts (orthologs) [7,8,9]. This genetic similarity makes zebrafish an excellent model for studying gene function and modeling human diseases, including PTSD.

In addition to their genetic advantages, zebrafish are becoming increasingly popular for anxiety and stress research because they exhibit a reliable and easily measurable cortisol stress response [4]. While zebrafish do not experience PTSD in the same manner as humans, their stress responses make them a valuable tool for studying the mechanisms underlying PTSD and testing potential treatments [10]. One of the key advantages of using zebrafish in this context is the optical transparency of their embryos and larvae, which allows researchers to observe neural activity and developmental changes directly and in real time, particularly in response to stressors during early life stages [11]. Moreover, the brain’s neuronal activity can be continuously measured not only in fixed individuals but also in freely moving zebrafish providing a great advantage for investigating complex behavioral reactions to stressors [12].

Both behavioral changes and an underlying (epi)genetic basis of reactions to various stressors are extensively studied in zebrafish. Acute stress in zebrafish can be induced by altering environmental factors such as air exposure, changes in water pH, salinity, temperature, crowding, handling, or exposure to bright light [2,13]. Psychological stressors, including social isolation and exposure to unfamiliar environments, may activate a stress response system in zebrafish leading to changes in behavior, gene expression, and proliferation of specific brain cells [14,15]. Nutritional stress also impacts growth, reproduction, and metabolism, resulting in behavioral and molecular adaptations [2,16,17]. Prolonged exposure to the stressors can lead to chronic stress, which manifests in persistent behavioral and molecular changes [2,16]. Other methods of inducing anxiety in zebrafish include exposure to alarm substances released by conspecifics and net chasing [2,18]. In response to these stressors, zebrafish may display anxiety-related behaviors such as increased locomotion, erratic swimming, freezing, avoidance of bright areas, memory deficits, and reduced cognitive function. The hypothalamic–pituitary–interrenal (HPI) axis in zebrafish is activated under these conditions, leading to cortisol release, which mirrors the human stress response and further supports the use of zebrafish as a model for studying PTSD and other neuropsychiatric conditions [2].

Building on recent advances in the field, we have conducted and recently published a comprehensive systematic review analyzing 40 zebrafish studies that employed a range of stress paradigms to induce PTSD-like phenotypes [19]. That review identified 14–15-day chronic unpredictable stress (CUS/UCS) protocols as the most effective and reproducible for modeling core PTSD dimensions, including anxiety-like behavior, cortisol dysregulation, and neuroinflammatory gene activation. However, numerous alternative paradigms—including acute, social defeat, and chemically induced stress models—remain widely used in the literature, often with inconsistent validation or unclear translational relevance. The current manuscript aims to critically contextualize and synthesize the diverse experimental strategies used to model PTSD in zebrafish, highlighting both validated approaches and those requiring refinement. We provide a comprehensive framework for understanding the comparative strengths, weaknesses, and future directions for zebrafish-based PTSD research.

## 2. (Patho)physiology of Stress Response in Zebrafish

The zebrafish (*Danio rerio*) serves as a valuable model for understanding stress response, particularly in the context of neuropsychiatric research, including studies on PTSD. Similarly to other vertebrates, zebrafish possess a well-conserved stress response system regulated by the hypothalamic–pituitary–interrenal (HPI) axis, which is functionally comparable to the hypothalamic–pituitary–adrenal (HPA) axis in mammals [20,21]. This system is crucial for the physiological and behavioral adjustments zebrafish make when they encounter stressors.

When zebrafish are subjected to a stressor, the stress response begins with the central nervous system’s detection of a perceived or real threat. This triggers the sympathetic nervous system, leading to the activation of chromaffin cells through cholinergic receptors. This immediate release of catecholamines prepares the fish for rapid action by increasing circulating levels of these hormones, which is essential for the immediate “fight or flight” response [22].

Cortisol, the primary glucocorticoid in zebrafish, is a key hormone in the stress response but its fluctuations are not always linked to negative stress. Variations in cortisol levels can occur due to normal physiological processes such as circadian rhythms, reproductive cycles, sex differences, and stages of maturity [23]. In some situations, these changes are associated with positive stress, or eustress, which can trigger beneficial activities like increased alertness, foraging, or reproductive behaviors [24,25]. Therefore, distinguishing between normal baseline cortisol levels and those elevated due to stress is essential for understanding how cortisol variations reflect either homeostasis or a response to stressors. For example, in certain fish species, seasonal shifts in cortisol levels are observed, particularly in relation to reproduction [18,26,27]. Interestingly, while female cortisol levels exhibit these seasonal changes, male cortisol levels do not show similar periodicity, though both sexes have elevated cortisol during peak reproductive periods [28]. This suggests that cortisol plays a dual role in both the stress response and reproductive regulation.

Cortisol is released with a delay compared to catecholamines. The process begins with the release of a corticotropin-releasing hormone (CRH) from the hypothalamus, which then stimulates the anterior pituitary gland to secrete adrenocorticotropic hormone (ACTH). ACTH travels through the bloodstream to the interrenal cells in the kidney, where it promotes the synthesis and release of cortisol. The dynamics between CRH and cortisol are tightly coordinated, and this finely regulated interplay governs how behavioral response in zebrafish is related to the nature and extent of stress [29,30]. Typically, cortisol levels peak within 30 min to one hour following exposure to a stressor, although this timing can vary depending on species, genetic background, environmental conditions, and stage of development [3,30]. For example, in zebrafish embryos, stress does not induce increased levels of catecholamines, as well as cortisol, in 48 h post-fertilization but induces a manifold increase in the levels at 96 h post fertilization [31].

Recent studies have provided further insights into the dynamics of cortisol release in zebrafish in different stress conditions. Zebrafish exhibit a rapid and sustained elevation in cortisol levels following acute stress, with cortisol levels beginning to rise around 15 min post stress and returning to baseline approximately 2 h later [32]. This rapid cortisol peak differs from what is observed in other fish species, where cortisol levels typically peak between 30 min and 4 h after the onset of stress [33]. Moreover, elevated cortisol response in zebrafish is observed under chronic high temperature conditioning, typical for their laboratory housing, compared to the cooler environments of some other freshwater or marine fish species [34].

Accurate quantification of stress-induced changes in cortisol levels in zebrafish, especially in larvae, is needed to follow the development of sustained psychophysiological disorders mimicking PTSD in humans [35]. In this context, it should be kept in mind that in humans, the trauma itself does not directly affect the levels of cortisol and catecholamines (often measured in urine samples) [36]. Instead, the PTSD is associated with higher catecholamine levels and not with sustained changes in urinary cortisol levels. In contrast, healthy human individuals display a manifold increase in cortisol levels in the stressful period, if compared to the stressless period [37]. In addition to these cortisol dynamics, the molecular response to acute stress in zebrafish involves a temporal pattern of changes in the expression of key stress-related genes. For example, brain CRH levels increase rapidly, approximately 15 min after stress exposure, corresponding with a sharp rise in CRH expression in the preoptic area [38]. POMC (pro-opiomelanocortin) transcripts, which are involved in the production of ACTH, reach their peak at 30 min post stress, indicating a faster response compared to the previously reported 60 min for pituitary mRNA levels [38]. Additionally, glucocorticoid receptor (GR) mRNA levels show a peak at 15 min post-stress, further illustrating the swift molecular response to stress in zebrafish [32]. It is interesting that in normal animals some genes are then under expressed in late post stress, indicating their dual role in the control of whole-body cortisol levels, i.e., in the initial rapid rise and then in the recovery to the base levels [38]. Notably, in a knockout model of zebrafish with a functionally inactive hsd11b2 gene—which is responsible for expression of corticosteroid 11-β-dehydrogenase isozyme 2, crucially involved in converting active cortisol to inactive cortisone—an acute stressor induced prolonged synthesis and release of cortisol, and therefore augmented the stress reaction, both in larvae and adult zebrafish [3]. In contrast, chronic glucocorticoid exposure in the early-life period blunts acute elevation of cortisol in stress conditions [39]. This indicates the crucial role of certain genes in the control of cortisol dynamics and its implication in the stress response.

The intensity of the cortisol response is influenced by several factors, including the developmental stage of the fish [21,40,41]. Younger fish often exhibit heightened stress responses during critical developmental periods, such as metamorphosis [42,43]. Moreover, if adult parents have been stressed before conception, the offspring expresses higher susceptibility to stress [44]. Additionally, genetic factors, such as differences between strains or stocks within the same species, and environmental factors, including temperature, water salinity, light conditions, and even the color of the tank background, can significantly impact the stress response [20,45,46,47,48,49]. Internal factors, like nutritional status, food contamination, and the presence of diseases, mediate further modulation of how zebrafish respond to stress via different mechanisms like oxidative stress and altered mRNA expression [17,50,51,52]. Surprisingly, cortisol release to acute stress was lower in adult zebrafish housed in isolation compared to the animals housed in groups of 10 [53]. This finding demonstrates that the extent of stress can be modulated by chemical or behavioral transmission in the media occupied by socially grouped fish—it imitates how stress can be transmitted intrasocially. However, if zebrafish were developed in life-long isolation, the baseline level of cortisol, as well as cortisol response to acute and chronic stress, is not changed compared to the same animals developed in groups [54].

Behaviorally, zebrafish display a variety of stress-induced features, including increased movement, erratic swimming patterns, freezing, and avoidance of brightly lit areas. These behaviors can be quantitatively assessed using various experimental assays, such as the novel tank diving test, light/dark preference test, and open field test. These assays provide crucial insights into the effects of stress on zebrafish behavior, which can be extrapolated for better understanding how stress-related disorders are developed in humans.

## 3. Environmental and Developmental Factors Affecting Stress Response

Various environmental factors affecting stress response in zebrafish are summarized in Table 1. Environmental cues, especially light and temperature cycles, are crucial for synchronizing the internal biological clocks of zebrafish, which in turn regulate their stress responses. These external cues, known as “zeitgebers”, help align the circadian rhythms of the fish with their environment, influencing the HPI axis and the associated stress responses [23].

### 3.1. Abiotic Stress-Induced Factors: Light Cycle and Circadian Rhythm

In zebrafish larvae, the establishment of behavioral rhythms is heavily dependent on light/dark (LD) cycles. Research has shown that larvae raised in constant darkness fail to develop proper circadian rhythms, whereas those exposed to LD cycles display rhythmic behaviors earlier, particularly under blue light compared to white or red light [55]. Proper exposure to these cycles is critical during early development. Larvae exposed to only a few LD cycles before being shifted to constant darkness demonstrate significantly reduced rhythmicity [61]. If a rhythm of light/dark cycle is altered against the normal rhythm, 8 to 15 days-post-fertilization zebrafish larvae show deficient total activity compared to the larvae subjected to the normal rhythm [62].

Moreover, the wavelength of light plays a significant role in the development and behavior of zebrafish larvae. Blue light has been shown to be more beneficial for the development and survival of zebrafish larvae compared to red light. Larvae exposed to blue light conditions develop behavioral and clock gene rhythms more quickly than those exposed to red light [63]. This phenomenon has also been observed in other species, where blue light can induce a shift in activity patterns during critical developmental stages [56]. The timing of stress exposure within the daily cycle is also critical. Nocturnal species typically exhibit stronger stress responses during daylight hours, while diurnal species, such as zebrafish, are more reactive to stress at night [23]. Additionally, melatonin, a hormone that regulates sleep and circadian rhythms, reduces both their locomotor activity and cortisol levels [64] and protects against neurological oxidative stress, improving behavioral parameters and memory [65]. On the other hand, acute and chronic stress inhibits the production of melatonin affecting its nocturnal level and subsequent anti-stress effects [66]. This highlights the intricate relationship between environmental light conditions, circadian rhythms, and the physiological stress response.

Proper exposure to light is essential for normal growth and survival of zebrafish larvae. Studies have shown that larvae raised in constant darkness fail to establish normal locomotor activity rhythms and exhibit lower overall activity levels [55]. Additionally, larvae that remain in darkness beyond critical early developmental stages often do not survive past 18 days post hatching. However, transferring these larvae to a regular light/dark cycle at 5 or 10 days after hatching significantly improves their survival rates, emphasizing the critical role of light in early larval development [55]. This is consistent with findings that the number of LD cycles to which zebrafish embryos are exposed directly affects the amplitude and timing of their activity rhythms. Larvae that experience only one or two LD cycles before being moved to constant darkness exhibit fewer circadian rhythms compared to those exposed to more cycles [61].

The circadian rhythms established during the larval stage continue to influence stress responses in adult zebrafish. Even in constant environmental conditions, adult zebrafish maintain strong circadian rhythms in their locomotor activity, which are significantly shaped by previous light/dark cycles. The peak of their activity typically occurs during the subjective day, as determined by prior light exposure, with a consistent free-running period of about 25.6 h [58]. This persistence of circadian rhythms under constant conditions indicates that zebrafish possess an intrinsic circadian clock. It is therefore an excellent model for studying the genetic and environmental factors that affect these rhythms. Molecular mechanisms of endogenous circadian rhythms as well as certain genetic mutations that alter the circadian period may be potentially discovered in zebrafish-based models which is translationally important for human disorder studies [67,68,69].

### 3.2. Abiotic Stress-Induced Factors: Thermal Stressing

Both heat and cold environments are abiotic stress-induced factors in zebrafish. The relative simplicity for their change and control makes them useful factors in studies related to stress outcomes.

Heat stress induced an increase in cortisol levels in zebrafish embryos between 1 and 4 days post-fertilization (dpf), as well as in 6 dpf larvae [70,71]. However, inconsistence is present regarding the expression of heat shock proteins (HSPs). For example, HSP70 was unaffected by the acute heating in zebrafish embryos [70]. In contrast, HSP70 and HSP90aa1.1/1.2 were elevated in both wild-type and corticotropin-releasing hormone receptor 1 (CRHr1)-deficient zebrafish larvae subjected to a 5 °C increase for 60 min [71]. Furthermore, heat affected the expression of various genes involved in metabolic activity and functioning including sugars and amino acids [70]. If zebrafish embryos were exposed for 24 h to fluctuating heat stress, this induced accelerated growth and activity as well as altered production of certain molecular markers like interleukin-1β. Interestingly, the embryos that are not directly imposed to temperature fluctuations but placed into the media where other individuals were previously imposed to these fluctuations, developed similar changes compared to those individuals [72]. Such findings illustrate well the possibility for stress propagation between conspecifics [70].

In addition to changes in expression of stress-related molecular factors, a short single heat shock may result in specific alterations in the molecular mechanisms of DNA damage repair, with distinct effects in zebrafish embryos at different post-fertilization stages [73]. The direction of alterations in DNA damage repair displayed dependence on both post-fertilization stages and the extent of heat stress in the zebrafish embryos [74].

Cold stress increases shoaling in zebrafish [57]. If applied during embryo and larvae development, it irreversibly damaged normal social behavior and decreased social contacts [75]. The alterations may be related to the damaged gene expression via affected miRNA functioning as it has been shown to be involved both in cold stress responses and, in particular, to the cold tolerance developed in zebrafish larvae [76]. Under lethal cold stress at 10 °C, the extensive transcriptomic analysis in 96 hpf zebrafish larvae revealed that some genes (protein kinases and phosphatases like trib3 and dusp5) change their expression level in a high correlation with cold-induced damage [77]. A large number of genes with highly distinct functionality (e.g., regulation of neural tissue development, sensing of temperature, production of cholesterol, and cell apoptosis) were detected as downregulated in cold stress [78]. Other studies showed that if zebrafish larvae have non-functional transmembrane proteins Tmem39b or Tmbim3a/Grinaa, the key proteins involved in cell autophagy and endoplasmic reticulum stress response, their resistance to cold stress and survival is significantly reduced [79,80]. Of note, the negative impact of cold-induced transcriptional alterations may be partially reduced if zebrafish larvae were initially pretreated by mild hypoxia [81]. Moreover, some genes are co-induced by cold and hypoxia, meaning the presence of a hypoxia-related mechanism protecting against cold injury. Moreover, zebrafish larvae have a rapidly (in hours) established mechanism preventing the negative outcomes of oxidative stress induced in cold stress [82,83].

In adult female zebrafish, cold stress at 10 °C or 18 °C for 48 h induced substantial alterations in numerous ovarian miRNA-mRNA, implying the role of this mechanism in further stress-induced effects in the progenies [84]. The spawning of female zebrafish has been shown to be temperature-dependent having a critical temperature when the spawning is extremely diminished [85]. Moreover, it was regulated by several hormones including insulin-dependent signaling. Also, in another recent study, it was observed that bile acids content is significantly elevated in the ovarian tissue of zebrafish subjected to acute cold stress with temperatures as low as 13 °C [86]. Cold stress also reduces aggression, as evidenced by decreased fighting behavior, and impairs learning ability, although memory remains unaffected [57]. It is therefore clear that stressing by cold operates via multiple molecular pathways.

### 3.3. Age-Related Differences in Stress Responses

Age is another critical factor influencing stress responses in zebrafish, paralleling observations in mammals. Zebrafish, like other species, undergo age-related changes that affect their physiological and behavioral responses to stress [2,43]. For instance, zebrafish become young adults at 3 months, are considered old at 30 months, and have a typical lifespan of about 4 years [87]. This life cycle gives zebrafish a relatively longer period of mature adulthood compared to mammals, potentially making them more resilient to stress during this phase of life. Interestingly, acute stress does not alter anxiety-like behavior and whole-body cortisol levels in young zebrafish, but it does in adults [2]. On the other hand, behavioral changes differ if young and older animals are subjected to the same chronic stress [43]. Compared to aged animals, younger animals display much greater alterations in anxiety-like responses to stress under chronic conditions.

Anxiety-like responses, as tested by a novel tank diving test, social interaction test, and shoaling behavior assessment, are more frequently observed in adult vs. young zebrafish that are both subjected to the same stressor [88]. Locomotor activity is also decreased with age, as evidenced by the increased immobility observed in 18-month-old zebrafish compared to younger individuals [2,89]. However, this effect was found to be strain-dependent implying involvement of some genetic background to the age-related changes in the behavioral responses to short and prolonged stresses [90]. Additionally, telomere length in both the brain and heart tissue of zebrafish is shortened in adult (18 months) vs. young (6–9 months) animals [89]. Moreover, the shortening is much greater in stressed vs. unstressed adult animals. These findings on stress-related effects on telomere length in zebrafish are in good accordance with the reported stress-related shortening in telomeres in young humans [91]. In addition, the level of oxidative stress in the brain tissue, as evaluated by the relative amount of oxidized proteins, is elevated in aged zebrafish [92], which may contribute to the age-specific neuronal functioning, especially to the response to stressors.

However, it should be noted that adulthood in zebrafish is relatively extended in proportion to other age stages (where the animals are considered young or old) compared to the same period in humans and other mammals [2]. This difference may be opportunistic for stress resistance in zebrafish compared to humans whose shorter adult period might render them more vulnerable to early-life stressors. This difference should be taken into account in the translational approaches between zebrafish and mammals.

### 3.4. Sex Differences in Stress Response in Zebrafish

Analysis of zebrafish stress responses reveals pronounced sexual dimorphism. Under basal conditions, female zebrafish display heightened anxiety-like behaviors—more bottom dwelling, increased freezing, and elevated resting cortisol and estradiol—whereas males exhibit greater exploration, reduced immobility, and lower endocrine stress markers [93,94]. Following acute stress, males mount larger cortisol surges than females, reflecting stronger HPI axis activation in males and possible estrogen-mediated attenuation in females [95,96]. Chronic or unpredictable stress paradigms further accentuate sex-specific coping: males show increased aggression and cortisol under UCS, while females remain behaviorally and hormonally stable, indicative of greater resilience [97]. Social and environmental modifiers—group housing, enrichment, and fluoxetine—attenuate stress responses in both sexes but disproportionately blunt male reactivity, narrowing sex differences [95,96].

At the molecular level, females express higher crha, crhr2, and actha but lower crhbp than males, correlating with elevated HPI reactivity; males’ increased CRH-binding protein may underlie their dampened neuroendocrine output [93]. Estradiol further modulates stress by suppressing brain serotonin and dopamine synthesis, linking sex steroids to both behavioral and endocrine phenotypes [93].

Moreover, sex-dependent transcriptomic profiling after social defeat reveals that “bold” male lines undergo extensive gene expression reprogramming—particularly in neuroimmune and synaptic plasticity pathways—whereas “shy” female lines exhibit minimal transcriptomic shifts but greater behavioral flexibility, underscoring divergent plasticity strategies [98]. Finally, assessments of nociceptive thresholds indicate no intrinsic sex difference in pain sensitivity, although environmental stressors can modulate nociceptive signaling in both sexes to a similar extent [99].

The sex-related differences in stress responses in zebrafish are shown in Table 2 that integrates and complements findings from multiple independent studies. Collectively, these zebrafish data mirror human patterns—where estrogenic regulation of the HPA axis and monoaminergic systems contributes to women’s higher vulnerability to stress-related disorders such as PTSD—and underscore the translational value of sex-specific stress models.

### 3.5. Stress Coping Styles

The variability in stress responses among different strains and species of zebrafish provides insight into the interspecies differences in stress coping styles. Zebrafish exhibit two primary coping styles: proactive and reactive. Proactive individuals tend to be risk prone and exhibit lower glucocorticoid responses, while reactive individuals are more risk-averse and display higher glucocorticoid responses [59]. In addition, proactive individuals are more aggressive and have lower frequency of shuttling between light and dark compartments but react swiftly to a stressor like acute water salinity [100]. Of interest, these coping styles are consistently stable in populations bred under certain circumstances indicating relatively little inter-individual variations. For instance, zebrafish selectively bred for reactive coping styles show significantly higher cortisol levels during the rising phase of the stress response compared to those bred for proactive coping styles. Furthermore, individual variation in cortisol levels and stress behaviors, such as depth preference in the novel tank diving test, are correlated only in the reactive individuals [59]. These differences in coping styles not only affect baseline stress responses but also significantly influence the acquisition and retention of fear-related memories. Thus, reactive subpopulations show more rapid learning and longer retention of fear memories compared to proactive individuals [60]. Also, the pro- and reactive phenotypes may be linked to the locomotor and metabolic activity (or vice versa). If zebrafish are bred in social defeat stress, they segregate to two subpopulations different in motility, boldness/shyness, and gene expression in the brain tissue [98]. Surprisingly, proactive shy fish were relatively resistant to the changes in gene expression in the brain. In contrast, reactive and low-mobile boldness-type fish had a much larger number of gene variations expressed in the brain.

In addition, specific zebrafish strains exhibit varying levels of stress sensitivity. Laboratory strains like the TAB line are generally less sensitive to stressors than wild strains but specific strains like Leopard and Albino present higher anxiety levels in behavioral tests [2,101]. AB strain zebrafish have higher basal whole-body cortisol levels and display lower inhibitory avoidance and shoal cohesion than TL zebrafish [102]. If compared to another teleost species, Japanese rice fish (also known as medaka, *Oryzias latipes*), which is also an emerging fish model to study behavioral responses, zebrafish display a set of quantitative and qualitative discrepancies, e.g., in the anxiety-like behavior tests (open field, diving, dark/light preference) and sociability tests (shoal cohesion and octagonal mirror test) [103]. This variability suggests that genetic and environmental factors significantly influence stress coping styles. Understanding these differences is crucial for correct interpreting stress-related studies in zebrafish and their applicability to other species, including humans.

### 3.6. Environmental Enrichment and Physical Exercise

Environmental enrichment (EE) refers to manipulations of the housing environment designed to enhance physical and social surroundings of laboratory animals. This approach has been extensively studied in rodents, where it has been shown to modulate susceptibility to stress, promote neuroprotection, enhance neurogenesis, and influence a range of behaviors [104]. EE is an important component of survival, growth, and development in zebrafish thus greatly necessary for taking into account in interpreting results obtained in various behavioral tests, especially if the effect of stress is studied [105]. This is primarily due to the direct and indirect links between welfare on the one side and anxiety and chronic stress on the other side [46]. For example, overcrowding due to the excessive housing density and reduced tank size significantly affects the performance of novel task test in adult zebrafish [13,48].

Recent studies have extended the investigation of EE to zebrafish, demonstrating its potential to mitigate the effects of chronic unpredictable stress (CUS/UCS) of a mild extent [106]. Specifically, EE administered for 21 or 28 days was found to attenuate the behavioral and physiological effects induced by CUS/UCS [104]. Zebrafish exposed to EE during this period exhibited reduced anxiety-like behaviors and lower cortisol levels compared to those not provided with enriched environments. Moreover, EE prevented the increase in ROS levels typically observed in zebrafish subjected to CUS/UCS, indicating a protective effect against oxidative stress [104].

These findings reinforce the idea that EE exerts beneficial neuromodulatory effects across species, including zebrafish. In particular, EE involves molecular pathways that reduce inflammation and oxidative stress while increasing cell proliferation and gene synthesis [107,108]. By enhancing the environment, EE reduces vulnerability to stress and mitigates its biochemical impacts, offering a promising avenue for improving resilience against stress-related disorders. The cross-species applicability of EE highlights its potential as a non-invasive intervention to counteract the negative consequences of chronic stress.

Physical exercise is another pre-treatment in zebrafish that may reduce stress-related effects. For example, physically trained zebrafish—i.e., exercised during 6 weeks in a waterflow compartment—exhibited reduced anxiety-like behavior both in the novel tank test and in the light–dark test compared to untrained group [109]. Forced swimming triggers a set of alterations in HPI axis that can further provide protective effect against stress [110]. It has been demonstrated that trained fish exhibit short- and long-term changes concerning energy supply mechanisms and resistivity to oxidative stress [111,112]. The attenuated oxidative stress in trained fish may have a stress-preventive effect as it has been shown that stress itself induces increased ROS production [113].

### 3.7. Feeding Regimens

A feeding regimen is an important factor that can affect both behavioral characteristics and response to stressors in larval and adult zebrafish [13,114]. It has been proposed that food itself and the feeding mode (in general, the daily caloric content of the food) are a new “zeitgeber” in studies related to the behavioral assessment in zebrafish [62]. However, the locomotor activity, which is used extensively as one of the measures in evaluation of stress, was shown to be non-related to feeding activity [115]. Instead, the locomotor activity was governed by the light–dark cycle per se, while the feeding activity was governed predominantly by the feeding time. In other words, if the feeding time was diurnal, the feeding activity was diurnal too, but the locomotor activity remained mostly nocturnal.

It has been shown that a high-fat diet resulted in cognitive impairment in adult zebrafish via the mechanisms found to be similar to mammals [51]. For example, certain genes related to the function of anti-oxidative stress systems were found to be downregulated while those responsible for apoptosis were upregulated in the fish fed by high-fat diet [51], which is relevant because neuropsychological disorders like PTSD or fear generalization have been linked to oxidative stress [113,116,117]. Therefore, the composition of nutrients in the feed may affect behavioral responses in zebrafish via oxidative stress-mediated mechanisms.

Finally, the beginning of feeding in larval zebrafish, which normally commences between 4 and 5 dpf, plays a role in the development of behavioral responses to potentially stressful stimuli. It has been found that the lack of feeding at 5th dpf does not produce any behavioral shifts [118]. In contrast, if no feed was provided at 6th and 7th dpf, the larvae displayed deficient avoiding of visual stimuli, decreased speed of swimming, and increased time spent in the rest state. Larvae which were fed normally were more socialized at 7th dpf (decreased thigmotaxis and inter-individual distance in the response to stressor) compared to the unfed larvae.

Therefore, the feeding regimen and amount must be considered important factors in stress-focused studies. According to a multi-laboratory study, it is highly recommended to follow standardized feeding regimen(s) in order to minimize possible inter-laboratory differences and interpretations in experimental findings related to acute and chronic stressing [119,120].

### 3.8. Social Interactions

Social interactions play a crucial role in modulating stress responses in zebrafish, as evidenced by studies showing the impact of conspecific presence during stress exposure. Zebrafish that were allowed to remain with conspecifics during stressful events exhibited reduced anxiety-like behaviors and engaged in more frequent social interactions compared to those that were isolated during stress exposure [88]. This suggests that the social environment can significantly buffer the negative effects of stress, highlighting the protective role of social support in reducing stress-induced behavioral changes [121].

Acute stress affects social behavior in zebrafish in an intensity-dependent manner [121]. Lower levels of stress primarily disrupted social maintenance behaviors, such as the ability to maintain social bonds and interactions with other zebrafish. In contrast, higher stress intensities impacted the zebrafish’s willingness to initiate contact with conspecifics. This gradient effect indicates that while mild stress may selectively impair ongoing social interactions, more severe stress can broadly suppress both the initiation and maintenance of social behavior. In addition, as different zebrafish strains express dissimilar extent of social approach [121] and a level of anxiety-like behavior [101], this should be taken into account in the designing of study.

Yet, a very important consideration should not be avoided. Zebrafish are socially organized animals which often build hierarchical communications, especially in small groups [122]. If same-sex zebrafish are housed in pairs, in most of the pairs there is remarkable dominant–subordinate behavior [123,124]. This dominant–subordinate axis is constituted by prominent molecular and genetic differences between the counterparts, e.g., in the level of plasma cortisol and expression of glucocorticoid receptor gene [125]. Importantly, this difference was demonstrated for both sexes. Of note, fish experiencing transition in hierarchical rank develop bodily changes as rapidly as within minutes, including level of gene expression in the brain and circulating hormones in the blood [126]. Specific molecular and genetic pattern can, in turn, either diminish or augment the susceptibility of dominant/subordinate individual to a stressor. Indeed, it is known that subordinates express elevated anxiety-like behavior compared to the dominating counterparts in almost all vertebrates [124]. Further studies in zebrafish may help clarify this specific issue and its importance for developing PTSD.

### 3.9. Learning Capabilities

Cognitive domains such as learning, memory, and recognition are central to understanding how trauma-related disorders like PTSD develop and persist. In humans, PTSD is not only characterized by fear responses and hyperarousal but also by maladaptive memory formation, fear generalization, and impaired extinction learning [127]. Understanding the learning capabilities of zebrafish is crucial for assessing the translational validity of stress models.

Learning in zebrafish, as in other animals, can be broadly categorized into associative and non-associative types [4]. Associative learning involves actively forming connections between two or more stimuli, leading to changes in behavior based on acquired knowledge. Examples include classical conditioning, such as aversive or fear conditioning, operant conditioning, and social learning, where animals learn by observing others’ behaviors [128,129]. On the other hand, non-associative learning does not require explicit associations between different stimuli. Instead, it involves behavioral changes in response to a single stimulus, typically resulting from repeated exposure or intensity variations [4]. This more passive form of learning leads to short-term, stimulus-specific behavioral modifications like habituation, sensitization, perceptual learning, priming, and recognition memory [129].

Adult zebrafish have demonstrated capabilities for episodic memory, which involves recalling specific events and their context. Studies have shown that when zebrafish are presented with a familiar object in a new location within a familiar context, they spend more time in that area [4,130]. These findings suggest that zebrafish can remember not only what they encountered but where and when it occurred. Furthermore, zebrafish also exhibit spatial learning, using visual landmarks and spatial cues to navigate their environment and remember the location of food or rewards in a complex maze, further underscoring their cognitive abilities and suitability for translational studies [128,131]. However, larval zebrafish display less advanced learning capabilities compared to adult individuals, and their use in corresponding studies should therefore be approached with care [13].

Furthermore, aging affects cognitive abilities of zebrafish, perhaps in a similar way to humans. Young zebrafish, aged before 1 year, do not display deficits in spatial and associative learning but animals aged 2 years or more do have such deficiency [92]. The zebrafish is therefore a good model for simulating various degenerative processes in neurons and neuronal tissue resulting in cognitive and neuronal dysfunction typical for some elderly human individuals, e.g., Alzheimer’s and Parkinson’s diseases. The recent advancements in this topic are discussed thoroughly in other reviews [132,133,134]. In addition, zebrafish are extensively used in models for studying learning and memory processes, in particular under drugs and substances targeting brain tissue [135].

These findings reinforce the importance of zebrafish as a model organism not only for studying the physiological and behavioral consequences of stress but also for dissecting the cognitive processes involved in trauma-related memory formation and extinction. Given that PTSD involves dysregulated fear learning, impaired memory extinction, and cognitive inflexibility, the ability to study these processes in zebrafish—including the effects of age, early life stress, and pharmacological interventions—enhances their relevance in PTSD research. Integrating cognitive assessment with stress paradigms may yield deeper insights into individual susceptibility, recovery potential, and treatment responsiveness in zebrafish-based PTSD models.

## 4. Standard Tests Used to Measure Stress Response in Zebrafish Studies

### 4.1. Behavioral Assessment

Before we step into the focused discussion of various PTSD models in zebrafish studies, standard methods and tests used to quantify and qualify the response to stress and stress-induced impairments should be briefly discussed. In zebrafish research, the novel tank diving task (NTT) and the light–dark tank test (LDT) are commonly employed to study anxiety-related behaviors [4,6,109,136]. These assays are particularly effective in evaluating how zebrafish react to stress, especially when introduced to new or unfamiliar environments, which typically elicit anxiety in these fish [4].

The NTT leverages the natural tendency of zebrafish to seek safety in the depths of an unfamiliar environment. When placed in a new tank, zebrafish usually dive to the bottom and stay there until they feel secure enough to explore higher regions. This test allows researchers to assess anxiety by observing behaviors such as the time it takes for the fish to swim to the upper parts of the tank, the frequency of transitions to the upper half, and the presence of erratic movements or freezing episodes [137,138]. Increased time spent at the bottom, fewer transitions to the upper areas, and more frequent erratic or freezing behaviors are interpreted as signs of elevated anxiety [139,140]. Another physiological measure has been recently proposed—bioelectrical signals called ventilator activity—allowing more precise characterization of emotional and behavioral responses of zebrafish to a novel environment [141,142]. Using these biosignals simultaneously with video-tracked swimming activity provides a better assessment of anxiety and fear behavior [141]. This tool was shown to be valuable in the analysis of fear/anxiety in zebrafish, allowing the discrimination of at least three emotional states related to norm and anxiety-like behavior [143].

The LDT, on the other hand, measures the zebrafish’s preference for either the illuminated or dark sections of the tank, reflecting a behavior known as scototaxis. This behavior, where zebrafish show a preference for dark environments, is thought to be an evolutionary adaptation to avoid predators. In this test, higher anxiety levels are indicated by a greater preference for the dark areas over the light ones [4]. Additionally, the dark–light transition test is used to evaluate changes in the zebrafish’s movement patterns when the lighting in the tank suddenly changes, providing further insights into how anxiety affects behavior.

In addition to the NTT and LDT, other measurable characteristics like erratic movement and freezing duration are used for the assessment of stress in zebrafish [144]. The characterization of such movement now is largely based on a real-time high-precision 3D reconstruction of swim trajectories with a subsequent analysis of several properties like prevalence in swimming in certain tank compartments, smoothness of swimming speed, angle of turn, etc. [145,146,147]. For example, the automated video tracking tool is successfully used for phenotyping individual zebrafish in response to various CNS-targeted drugs [145,148] as well as in response to environmental cues like visual stimuli [147].

Of note, the utilization of advanced computational methods for the precise evaluation of zebrafish behavioral responses to different tests allowed for revealing individual correlations between certain behavioral variables. It was revealed by the help of machine learning that different variables related to NTT were weakly correlated to variables related to open field test but were in high correlation between themselves [149]. Moreover, sex differences were found in this regard, with stronger correlation in male vs. female individuals. In addition, swimming- and exploring-related variables were weakly interdependent, according to machine learning-based analysis [149].

These behavioral assays are fundamental tools in the study of anxiety in zebrafish, offering a quantitative way to measure anxiety-like behaviors under different conditions. These methods are essential for advancing our knowledge of stress and anxiety mechanisms in vertebrates, with implications for broader applications in neuroscience and behavioral research.

### 4.2. Biochemical and Molecular-Genetic Assays

In addition to the battery of behavioral tests, molecular markers exist that allow the assessment of stress response in zebrafish studies and validation of PTSD models. These include the markers related to HPI axis or neurotransmitters, and we provide here a brief discussion on their implications in stress response studies.

#### 4.2.1. Hypothalamic–Pituitary–Intervertebral Axis Assessment

Corticotropin-releasing hormone/factor (CRH/CRF) is a key peptide hormone initiating the HPI axis response in fish and plays an essential role in stress regulation [150]. As the initiating factor of the HPA axis (or HPI axis) in fish, it plays a pivotal role in the stress response. In zebrafish, CRH is secreted by neurons in the neurosecretory preoptic area (NPO), functionally analogous to the mammalian paraventricular nucleus (PVN) [151]. Unlike mammals, most teleosts possess two CRH genes (crha and crhb) due to genome duplication; however, zebrafish uniquely lack this duplication, retaining only a single CRH gene [152,153]. Crhb is regarded as the functional ortholog of the mammalian CRH gene [152]. The CRH system in fish includes signaling peptide urotensin I that promotes intracellular cell signaling via specific G-protein coupled receptors and a shared binding protein that modulates these receptors [154]. This complex interplay mediates CRH-related endocrine regulation but is also mediating locomotion and behavioral changes induced by the stress. In larval zebrafish subjected to acute thermal stressing, CRH was found to be mediating both hyperactivity in locomotion and anxiogenicity of the stress response [71]. A study by Vom Berg-Maurer et al. (2016) demonstrated that CRH neuron activity in zebrafish is finely tuned to stress intensity, indicating its relevance as a molecular marker of stress reactivity in *Danio rerio* [155]. This is consistent with findings in rodent models of anxiety–depressive disorders [29,156,157] and in humans with major depressive disorder [158].

Adrenocorticotropic hormone (ACTH) is a peptide hormone synthesized from the prohormone proopiomelanocortin (POMC) in the anterior pituitary [159]. ACTH binds to melanocortin 2 receptor (mc2r, the ACTH receptor) in the interrenal tissue (analogous to the adrenal cortex in mammals), promoting the synthesis and secretion of glucocorticoids—thus forming a critical component of the HPI axis [160]. Studies on zebrafish larvae have shown that a rapid motor response to acute stress requires cortisol secretion through mc2r and depends on the canonical type 2 glucocorticoid receptor (GR) encoded by nr3c1 [161]. Consequently, the genetic knockout of the nr3c1 reduced locomotor activity in basal settings, both in light and dark compartments, in larval zebrafish [162]. Interestingly, rapid adaptation to environmental lighting conditions did require expression of mc2r but only if the lighting regimen was not prolonged and it was more effective in dimmer conditions [162]. Zebrafish produce ACTH exclusively from a single POMC gene due to a nonfunctional duplication [153]. The zebrafish lacking POMC expression demonstrate a steadily reduced level of cortisol release in response to stress as well as showing remodeled GR responsiveness to the stress mediators, essentially due to the lack of POMC-mediated signaling [163].

Cortisol is the primary glucocorticoid in teleosts [164] and a widely used biomarker of physiological stress [113,165,166,167,168,169]. In zebrafish, acute stress induces a cortisol surge peaking around 15 min post exposure and resolving within two hours [32]. In contrast, chronic or excessive stress can cause sustained cortisol elevation, indicating maladaptive HPI axis activation [167], which also happens in mammals under conditions of chronically elevated stress hormones [170]. In addition, elevated cortisol levels mediate stress-induced deterioration via complex neuroinflammatory pathways [171]. Therefore, cortisol is not only the principal molecule mediating the stress response but also the key inhibitor of ongoing HPA/HPI axis activity [172].

Cortisol exerts its effects via glucocorticoid receptors (GRs) and mineralocorticoid receptors (MRs), which modulate stress adaptation across molecular, cellular, and systemic levels [4]. Disruptions in GR signaling are linked to deficits in learning and memory, as well as to various brain disorders [173]. Unlike most teleosts that express two GR isoforms (GR1 and GR2), zebrafish express only one GR gene, nr3c1, which yields two splice variants: GRα (cortisol-binding, transcriptionally active) and GRβ (lacking binding and transactivation functions) [153,174]. GRβ can inhibit GRα activity, as shown in in vitro assays [153,174]. It is therefore suggested that the ratio between GRa and GRb, but not the actual expression levels, plays the main role in the cortisol-mediated stress regulation [175]. Compared to both GR isoforms, MRs are high-affinity substrates for cortisol that are considered to be nearly saturated at basal levels of cortisol; so, MRs perform a distinct role in homeostasis and the partial buffering of excessive cortisol [176].

Ziv et al. (2013) reported that zebrafish gr^s357^ mutants with impaired GR function show elevated CRH, ACTH, and cortisol levels, along with heightened stress-induced behavior [177]. This supports the notion that disrupted GR transcriptional regulation contributes to affective disorder phenotypes [177,178]. GRα expression is prominent in brain regions involved in stress adaptation, social interaction, and sensorimotor control, emphasizing its conserved role in stress regulation [178].

GR mRNA levels in zebrafish increase within 15 min of acute stress and normalize within 30 min [32]. Xin et al. (2022) further showed that early-life prednisolone exposure alters adult stress-related behavior through aberrant nr3c1 methylation and expression [179]. Excess early-life cortisol may epigenetically suppress GR gene expression, increasing stress vulnerability in adulthood [180]. Moreover, stress-induced cortisol release is blunted in zebrafish larvae lacking the transcription factor rx3, which disrupts pituitary-interrenal development [181]. Interestingly, these mutants still exhibit stress-typical behaviors, suggesting that some stress responses may occur independently of cortisol signaling.

#### 4.2.2. Neurotransmitters

Monoaminergic neurotransmitters are key regulators of alertness, mood, and emotional states [182]. Their dysregulation is implicated in the pathophysiology of long-term sequelae following traumatic experiences. Zebrafish possess neurotransmitter systems—including dopaminergic, serotonergic, noradrenergic, glutamatergic, and purinergic—that closely parallel those of mammals in terms of function and anatomical homology, albeit with a simpler organizational structure [183,184]. Given this evolutionary conservation, studying these systems in zebrafish PTSD models holds significant translational relevance.

Dopamine (DA) and noradrenaline (NA) are the main catecholaminergic neurotransmitters in zebrafish. The dopaminergic system is developed fully on 96 h post fertilization, with dopamine being a primary precursor for other catecholamine neurotransmitters like noradrenaline [185]. NA plays a vital role in autonomic function and stress reactivity, and dysregulation of the NA system is a central feature of PTSD pathophysiology [186]. The populations of dopaminergic neurons and noradrenergic projections mirror those in mammals [185,187]. In zebrafish, the noradrenergic system is established early in development—NA-producing neurons are detectable at 16 h post fertilization and remain stable into adulthood [188]. It has been reported recently about elevated NA levels in zebrafish exposed to traumatic stress, accompanied by heightened anxiety and impaired risk assessment—findings consistent with mammalian models and human clinical data [189,190].

Dopamine plays a central role in learning, memory, motivation, motor control, feeding, and fear regulation [191]. As a precursor of both noradrenaline and adrenaline, DA also contributes to the neuroendocrine stress response and PTSD etiology [192]. Although zebrafish lack midbrain dopaminergic nuclei typically found in mammals, they possess alternative dopaminergic populations, including a unique pretectal group [193], underscoring species-specific anatomical adaptations within a conserved functional framework.

Serotonin (5-HT) is a multi-functional monoamine, primarily active in the central nervous system, but also present in the gastrointestinal tract and platelets. It mediates both excitatory and inhibitory signaling, and regulates aggression, anxiety, cognition, mood, sleep, and memory [194,195]. Within the brain, 5-HT modulates defensive behavior by suppressing fight-or-flight responses via the periaqueductal gray (PAG), while concurrently promoting anxiety-related processes in the amygdala [196]. PTSD patients often present with diminished 5-HT levels, which are associated with increased aggression, impulsivity, depression, and suicidality [197,198]. Supporting this, Grigorios Oikonomou et al. (2019) demonstrated that genetic ablation of the raphe nuclei in zebrafish—key centers of 5-HT synthesis—leads to reduced sleep duration and depth, and impairs sleep homeostasis [199], paralleling sleep disturbances commonly observed in PTSD [200]. Also, an intense release of 5-HT is required for the synchronization of neurons in the dorsal pallium of zebrafish that substantiate the vigilant state of the animals—the behavioral marker of alertness and threat-preparedness [201].

The monoaminergic markers discussed above are vital for the development and validation of PTSD models in *Danio rerio*. However, the repertoire of relevant biomarkers extends further. Zebrafish express homologues of numerous clinically recognized PTSD-associated molecular targets, including components of the glutamatergic and GABAergic systems, the (anti)oxidative stress system, receptors for 5-HT, DA, and NE, brain-derived neurotrophic factor (BDNF), corticotropin-releasing factor (CRF), catechol-O-methyltransferase, neuropeptide Y, calcineurin, among others [6,193,202,203,204].

### 4.3. Advances in Optogenetics and Real Time Neuroimaging

Over the past year, zebrafish stress research has been transformed by two complementary technological innovations: optogenetic manipulation of the HPI axis and high-resolution functional neuroimaging. By driving expression of photoactivated adenylyl cyclase (bPAC) in interrenal steroid cells, investigators can now modulate endogenous cortisol on a millisecond timescale. Brief pulses of blue light evoke rapid cortisol spikes accompanied by increased larval locomotion, whereas prolonged illumination produces chronic hypercortisolemia and shifts in HPI feedback set points, thereby distinguishing non-genomic from genomic glucocorticoid signaling [205]. Extending this approach to pituitary corticotrophs via the POMC promoter has directly linked cAMP-driven ACTH release to downstream cortisol surges and stress-related behaviors in freely swimming larvae [206]. Moreover, chronic optogenetic elevation of cortisol throughout development—achieved with a StAR:bPAC-tdTomato transgene—induces lasting basal hypercortisolemia, blunted acute stress responses, and an HPI “programming” signature analogous to that observed after early-life adversity [39]. In the recently described Tg(star:bPAC 2A tdTomato)uex300 line, sustained early-life glucocorticoid overexposure drives rapid proliferation of hypothalamic progenitors and premature feeding but ultimately depletes progenitor pools, disrupts neuronal maturation, stunts growth, and compromises survival [207].

At the same time, genetically encoded calcium indicators such as GCaMP6 variants have become indispensable for monitoring neuronal and epithelial activity in vivo. Using Tg(HuC:GCaMP6F) larvae, two-photon imaging has revealed that acute chemical and electrical shock stressors elicit rapid calcium transients in area postrema noradrenergic neurons, driving heightened swim bouts; paradoxically, prolonged stress suppresses both behavior and calcium activity, an effect abolished by α-adrenergic blockade [191]. In a Carp β-actin:GCaMP6s line exposed to heat stress, transient calcium spikes in head and trunk epithelia peak at approximately 30 s; pharmacological inhibition of voltage-gated calcium channels, CaMKII, or heat shock factor 1 attenuates both the immediate calcium surge and downstream heat-shock protein induction [208]. More recently, imaging of Tg(gfap:jRGECO1a) larvae has shown that ketamine—but not selective serotonin reuptake inhibitors—elicits a sustained, α_1_-adrenergic-dependent glial calcium elevation in hindbrain astrocytes; this glial signal is both necessary and sufficient to suppress stress-induced passive behavior [209].

Despite these remarkable advances, truly brain-wide imaging modalities such as light-sheet or volumetric two-photon microscopy have yet to be applied to zebrafish stress paradigms, and fMRI remains impractical given the animal’s size and aquatic physiology. Likewise, high-speed voltage sensors (e.g., Voltron, ASAP1, zArchon1) capable of kilohertz-rate, single-cell recordings [210,211] have not been exploited in HPI axis studies. Photoconvertible reporters such as CaMPARI2 enable “freeze-frame” labeling of neurons active during defined stress episodes [211], and FRET-based chameleons afford ratiometric calcium measurements with minimal motion artifacts, yet both remain underutilized. Together, these optogenetic and neuroimaging innovations offer minimally invasive, reversible, and highly precise strategies for dissecting stress physiology at molecular, cellular, and circuit levels, and they chart a clear path for the future application of brain-wide and electrical-activity mapping techniques in zebrafish stress research.

## 5. Zebrafish Models for PTSD Research

Similarly to rodent models, zebrafish PTSD models are categorized based on the complexity and nature of the stressors, reflecting various facets of PTSD as seen in humans. According to the type of model, they can be classified as Predator Exposure Models, Conspecific Alarm Substance (CAS), Chronic Unpredictable Stress (CUS/UCS), Looming Dot Stimulus (LDS), Social Isolation Models, Pharmacological Stress Models, and Early Life Interventions (Table 3). This section critically examines the broader landscape of existing models. While some paradigms remain exploratory or show limited validity, they offer mechanistic or practical value and continue to shape experimental PTSD research.

Among other key features, whether a model operates in single or multi-stressor paradigm is a crucially important factor. Single stressor models in zebrafish involve the application of one acute or chronic stressor to induce PTSD-like symptoms. These models are particularly useful for dissecting the immediate and direct effects of a specific stressor, such as temperature fluctuations, physical restraint, or social isolation. For example, physical restraint can mimic aspects of the immobilization stress used in rodent models, leading to increased anxiety-like behavior in zebrafish [2]. Single stressor models provide a controlled environment for studying the basic mechanisms of stress response in zebrafish and can be directly compared to similar single-stressor models in rodents [212]. These models enable researchers to study behavioral changes, hormonal dynamics, and molecular pathways in a controlled and, in general, ethically acceptable manner. However, our review highlights that single-stressor models tend to lack long-term predictive validity and rarely capture the complexity of chronic trauma. Their outcomes are often transient, and their translational relevance to persistent PTSD symptoms—such as emotional dysregulation, hypervigilance, or cognitive impairment—remains limited.

First, some methods like electric shock may indeed raise ethical issues. Another limitation concerns translational challenges because the difference between zebrafish and humans in physiology and stress responses can affect the extrapolation of findings. Finally, stress intensity and the duration of a single stressor may not fully capture the complexity of PTSD, which often involves chronic and multi-faceted stressors. When utilizing single stressor models in zebrafish for PTSD research, it is essential to ensure standardized protocols for reproducibility and acknowledge the limitations in translating findings directly to human conditions. These paradigms are best applied to model discrete components of PTSD or to investigate stress thresholds and neuroendocrine reactivity in controlled settings.

Multi-stressor models, which involve exposing zebrafish to a combination of stressors over time, offer much closer approximation to the complex nature of PTSD in humans. These models simulate real-life scenarios where individuals are exposed to a series of unpredictable and varying stressors. Chronic unpredictable stress (CUS/UCS) protocols are commonly used, where zebrafish are subjected to different stressors such as unpredictable changes in water conditions, social stress, and predator exposure in a randomized sequence. This approach mirrors the multi-stressor models in rodents, where a combination of psychological, social, and physical stressors is applied to induce PTSD-like symptoms [15,113].

The CUS/UCS paradigms produce the most consistent behavioral, hormonal, and molecular changes, including anxiety-like behavior, sustained cortisol elevation, and pro-inflammatory gene expression [19]. These features closely mirror core PTSD dimensions in mammals and support the gold-standard status of CUS/UCS among zebrafish PTSD models. Notably, the predator exposure model in zebrafish, where the fish are repeatedly exposed to predator cues, has been shown to induce anxiety-like behaviors and relevant gene expression changes [213]. These behaviors parallel the fear conditioning and hypervigilance observed in rodent models exposed to predator scent or shock-pairing paradigms [214]. Zebrafish are highly social animals, and social stress models exploit this trait to study PTSD-like symptoms. Social isolation, a commonly used stressor, induces significant behavioral changes in zebrafish, such as increased anxiety and altered social interactions, which are relevant to the social withdrawal and relational difficulties seen in PTSD patients [14,97,213]. This mirrors the social defeat and social isolation models in rodents, where prolonged social stress leads to persistent changes in behavior, indicative of anxiety and depression [214,215,216]. Although social stress models show promise in capturing social dysfunction—a major PTSD symptom cluster—their long-term effects and molecular correlates are less thoroughly validated. Additional studies are required to elevate their translational utility in the impact of social environments on stress resilience or vulnerability.

The efficacy of pharmacological agents in modulating PTSD-like behaviors is another critical area of study in zebrafish models. Just as in rodent models, where the effects of SSRIs, anxiolytics, and other drugs are tested, zebrafish models have been employed to screen for potential therapeutic agents that can mitigate stress-induced behaviors and affect memory and learning capacities [4,14,217,218]. The use of zebrafish in high-throughput drug screening is particularly advantageous due to their small size and the ability to observe drug effects in real-time.

Whatever the nature of stressor, zebrafish PTSD paradigms can be further categorized based on the duration of exposure (chronic vs. acute or intermittent), the predictability of stress (predictable vs. unpredictable), and the strength of stressor. The number of simultaneously active stressors is considered as an additional powerful factor. This framework enhances the translational relevance of zebrafish models, aligning them more closely with established classifications in rodent PTSD studies.

Chronic stress paradigms involve prolonged or continuous exposure to stressors without recovery intervals, leading to sustained HPI axis activation, resource depletion, and adaptive fatigue. Examples of chronic stress in zebrafish include continuous predator exposure, prolonged social isolation, or sustained mild stressors such as temperature fluctuations or water quality changes. These models mimic the long-term nature of stress seen in disorders like PTSD and are particularly useful for studying stress-induced dysregulation of the HPI axis. Acute or intermittent stress paradigms involve either single or episodic stress exposure interspersed with recovery periods, simulating discrete traumatic episodes. In zebrafish, examples include periodic alarm substance exposure, episodic predator encounters, or randomized electric shocks. These models provide insights into how repeated acute stress can cumulatively affect behavior and physiology, mirroring the episodic nature of trauma experienced in PTSD.

Predictability is another critical factor influencing stress outcomes. Although zebrafish do not possess the same advanced predictive abilities as humans, they do exhibit a more rudimentary form of “prediction” through learned avoidance. For example, when shocks or other stressors consistently occur in a specific area, zebrafish reduce their time spent in that zone, effectively anticipating an aversive event. Under predictable stress, repeated cues (e.g., specific tank illumination before mild shocks or predator exposure) allow the fish to develop partial coping strategies or anticipate aversive events, often resulting in a more “training-like” or adaptive response. In contrast, unpredictable stress—characterized by random timing and diverse stressors such as abrupt predator cues, sudden net chasing, or alarm substance introduction—sustains heightened arousal and hypervigilance, more closely mirroring the persistent anxiety observed in PTSD.

Certainly, stressor intensity is one of the cornerstones in stress outcomes. It is clear that the stronger the stressor (irrelevant to its nature), the higher its psychophysiological effect on the individual. This key component of modeling PTSD must always be taken into account when evaluating the outcomes of the stress model, especially its relevance to the effects typical in human PTSD. As our analysis underscores, these classifications are not merely heuristic—they meaningfully impact the behavioral and molecular outcomes observed. The interplay between chronicity, unpredictability, and stressor intensity determines the extent to which a model approximates real-world PTSD trajectories.

**Figure 1:**
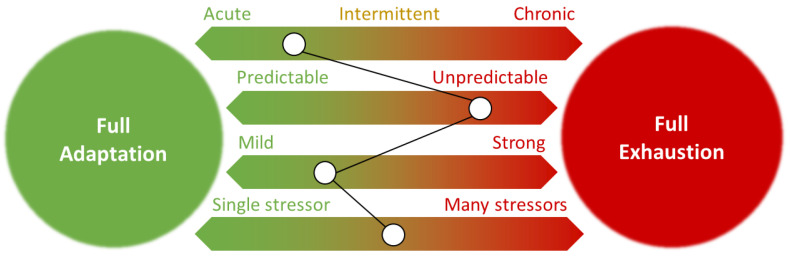
Simple graphical representation for a putative generalized pathological outcome of a certain combination of four key factors of stress: duration (acute, intermittent, or chronic), its predictability or unpredictability, the strength of the stressor (mild or strong), and the total number of simultaneously applied stressors. The outcome can be “adjusted” by selection of the desired level of factors (as in an equalizer) and therefore may range from full adaptation to stress to full exhaustion and death.

Finally, the stress outcome should differ in cases of single stressor vs. multiple stressors being actively applied at the same time. As we demonstrate in the corresponding section below, complex (multi-stressor) models of PTSD may produce a cumulative effect distinct in its features compared to the single-stressor effect. On the other hand, the number of simultaneous stressors itself is not a leading factor because both the type (quality) and the strength of stressor(s) affect. This is why it is more reliable, from our point of view, to consider the stress outcome as a functional output dependent on many inputs (Figure 1).

By integrating chronic vs. acute or intermittent, predictable vs. unpredictable, mild vs. strong stress, and single-stressor vs. multiple-stressor paradigms, zebrafish models allow researchers to investigate distinct yet interrelated aspects of PTSD. Mild stressor paradigms can be employed to induce primarily physiological (rather than overtly pathological) responses, providing insight into the organism’s adaptive reserves and liabilities in escaping or mitigating stress. In contrast, strong stressor paradigms often drive more severe neurophysiological exhaustion and imbalances in adaptive responses, thereby modeling pathological processes akin to those seen in human PTSD.

Although predictable paradigms commonly foster more “training-like” coping behaviors (since cues or patterns offer a limited anticipation of stress), it is important to note that repeated or prolonged exposure in such paradigms can still give rise to maladaptive responses if the stress becomes too intense or protracted. Conversely, unpredictable paradigms typically evoke persistent hypervigilance—one of the core features of PTSD—yet even under unpredictable conditions, zebrafish may exhibit partial coping strategies or transient forms of adaptation, reflecting the complexity of stress responses.

Meanwhile, chronic exposure reflects long-term resource depletion and “resilience fatigue,” mirroring the sustained allostatic load often observed in stress-related disorders. Acute or intermittent paradigms, with single or episodic aversive stimuli interspersed by recovery intervals, replicate the cumulative nature of trauma and underscore how repeated acute stress episodes can compound over time.

Taken together, this revised model taxonomy not only categorizes zebrafish PTSD paradigms, but also critically evaluates their translational weight. Overall, this categorization enables the modeling of diverse PTSD mechanisms in a systematic manner and supports translational research aimed at understanding and treating stress-related disorders. In the following subsections, we discuss each experimental model in terms of its biological validity, empirical support, and alignment with validated frameworks emerging from the literature and our own systematic review.

### 5.1. Electric Shock Models

#### 5.1.1. Application of Electric Shock

**Figure 2:**
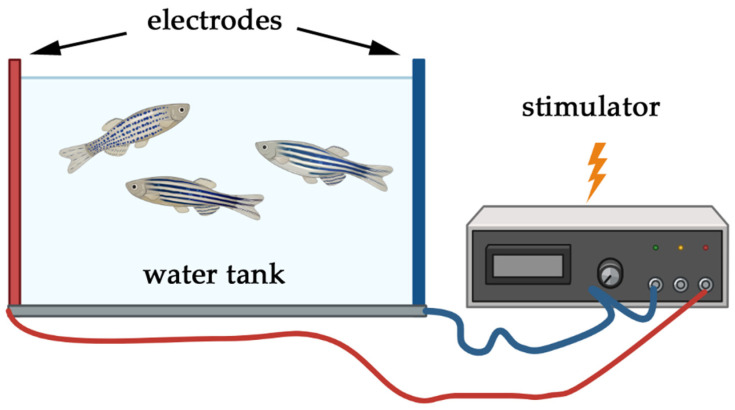
Simple representation of a setting typically used to implement an electric shock in zebrafish model of PTSD.

Electric shock models in zebrafish involve delivering controlled electrical stimuli to induce stress responses (Figure 2), providing important insights into the neural mechanisms of acute stress and fear learning. Although this approach is not as prevalent as in rodent research, it remains a valuable method for investigating discrete PTSD-relevant phenomena such as associative fear conditioning. These setups are primarily employed in acute, predictable stress paradigms, given that the stimuli can be precisely timed and consistently applied. Notably, shock intensity can be readily modified to range from mild stress to severe effects.

Several studies have explored zebrafish responses to mild electric shocks to model associative learning and memory processes. For instance, Yashina et al. (2019) [219] developed a conditioned place avoidance (CPA) paradigm using a shock of around 3–5 V in one compartment of a two-compartment tank. Zebrafish learned to avoid the compartment paired with the mild shock, demonstrating robust aversive learning [219]. Blank et al. (2009) adopted a similar approach in a single-trial inhibitory avoidance test, applying a 5 s mild shock to one compartment in a two-compartment bright–dark tank, thereby inducing rapid avoidance in less than 2 min [220]. Xu et al. (2007) reported that similar shock-based designs implicate various neurochemical pathways in zebrafish learning and memory [221].

Studies have also highlighted that contextual fear conditioning in zebrafish can be modulated by shock intensity. Furthermore, the extinction rate of this conditioning appears to be dependent on genetic background, indicating potential strain-specific differences [219,222]. Recent technological advances have facilitated high-throughput applications of electric shock in zebrafish larval assays. One example is a microfluidic platform equipped with a head immobilization feature that enables the detailed analysis of larval responses to electric fields, including direction, voltage magnitude, and habituation patterns [223]. Another innovation involves using electrode clips in a 96-well plate format to simultaneously track up to 80 larvae’s locomotor responses to electrical stimuli, significantly increasing potential throughput for screening applications [224].

Electroshock protocols can be optimized to minimize physiological damage and stress. For example, a brief shock at 3 V and 1 A for 5 s did not adversely affect adult zebrafish survival, muscle bioenergetics, or behavior under certain conditions [225]. In larval zebrafish, electrical stunning has shown promise as a rapid and reliable euthanasia method that quickly abolishes coordinated brain activity, aligning with growing efforts to refine the humane treatment of zebrafish in research [226]. Other recent work on larvae indicates that both noxious chemicals and electric shocks elicit similar changes in behavior: an acute increase in swimming activity followed by a decrease during prolonged exposure [227]. Neural correlates have been observed in the area postrema, suggesting the involvement of noradrenergic and dopaminergic circuits. Studies show that shock frequency and waveform characteristics also influence fear-related behaviors [228]. As variability remains high across protocols and platforms, future studies should aim to harmonize procedures to improve comparability and replicability.

#### 5.1.2. Relevance of Electric Shock Models to PTSD

Researchers use electric shock models to examine associative learning and fear conditioning under controlled conditions, mirroring single-event traumas rather than chronic stress syndromes. Such models in zebrafish have enriched our understanding of fear and avoidance behaviors, stress neurobiology, and gene–environment interactions that may contribute to PTSD-relevant symptom clusters. Experimental work using these paradigms has demonstrated that zebrafish subjected to electric shock often show elevated cortisol levels and increased expression of genes such CRF and GR, indicating activation of the HPI axis [229]. These biological changes reflect conserved components of the vertebrate stress response, although they do not alone replicate the full PTSD phenotype.

Associative learning studies in zebrafish have revealed that they rapidly form aversive memories linked to specific contexts or cues paired with an electric shock, while variations in shock intensity can alter acquisition and retention of fear. Fear extinction has been shown to differ across zebrafish strains, suggesting that genetic background can affect learning and memory processes [219,222]. Further research has established that electric shocks function effectively as a punisher of operant behavior, comparable to exposure to predator videos [230]. However, as highlighted by our systematic review, electric shock models primarily capture acute and controllable aspects of trauma exposure. While valuable for dissecting fear conditioning and acute endocrine responses, they lack the chronicity, unpredictability, and emotional complexity needed to develop PTSD in its full syndromic form. They are best suited for exploring neurobiological mechanisms of fear learning, stress hormone dynamics, and context-specific memory, providing a mechanistic bridge between basic stress biology and trauma-related research. The key advantages and limitations of the models are shown in Table 4. Yet, due to their limited ethological and etiological scope, they should not be considered a substitute for validated chronic stress models such as 14-day CUS/UCS, which better replicate persistent neuroendocrine and behavioral dysregulation.

### 5.2. Immobilization Stress Models

#### 5.2.1. Application of Immobilization Stress

**Figure 3:**
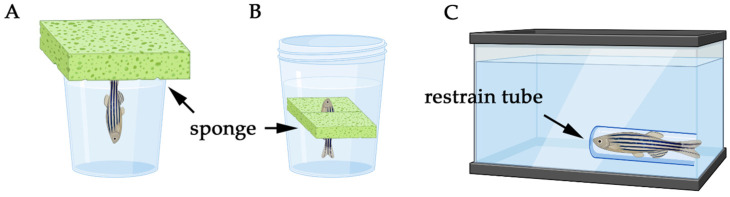
Different methods to immobilize a zebrafish in the model of stress-induced PTSD. (**A**) A fish is immobilized by fixing its tail into the sponge. (**B**) A fish is immobilized by inserting its body into the sponge. (**C**) A fish is restrained by putting it into a small container, like a tube.

Immobilization stress in zebrafish, often referred to as spatial restriction-based stress, is induced by confining the fish in small spaces, such as narrow tubes, or gently restraining them using mesh or sponge material (Figure 3). This method primarily limits the natural swimming behavior of zebrafish, leading to significant physiological and behavioral alterations. Depending on the application, this stressor can be either acute or chronic, as well as predictable or unpredictable. Predictable immobilization paradigms allow for the study of anticipatory anxiety and adaptive responses, whereas unpredictable immobilization introduces elements of heightened vigilance and fear responses akin to PTSD. Similarly, the intensity of immobilization varies depending on the duration—shorter durations induce mild stress, whereas prolonged immobilization leads to a more profound physiological response, including oxidative stress and neurotransmitter alterations.

Several studies have demonstrated the effects of immobilization on zebrafish physiology. Piato et al. (2011) implemented a restraint stress protocol in which zebrafish were placed in a plastic microtube (2 mL), with a small opening at both sides, to restrict locomotion for 90 min [113]. This exposure resulted in increased cortisol levels, decreased exploratory behavior, and enhanced thigmotaxis, indicative of heightened anxiety [231]. Restraint stress (RS) also led to decreased catalase activity and increased lipid peroxidation, reflecting oxidative damage, as well as increased non-protein thiol levels, suggesting a compensatory antioxidant response [231,232].

In addition to oxidative stress, immobilization stress significantly affects purinergic signaling and GABAergic neurotransmission in zebrafish brains. RS exposure for 90 min resulted in increased ATP hydrolysis, decreased cytosolic adenosine deaminase activity, and changes in the expression of genes involved in the adenosine signaling pathway. These molecular alterations align with findings in mammalian studies, where adenosine-mediated signaling is implicated in post-stress recovery mechanisms [231]. Furthermore, immobilized zebrafish exhibit a marked decrease in gamma-aminobutyric acid (GABA) release and reduced c-fos protein expression in the telencephalon, a brain region associated with stress reactivity [233]. This decrease in GABAergic inhibition may contribute to the anxiety-like behavior observed following acute restraint stress, reinforcing the model’s potential relevance to PTSD research.

Notably, even short-term confinement stress induces measurable neurophysiological effects in zebrafish. A 15 min acute confinement (netting) stressor, followed by behavioral testing in the open field and light/dark preference tests, revealed significant metabolomic changes. Alterations were noted in multiple pathways, with elevated levels of alanine, taurine, adenosine, creatine, lactate, and histidine, suggesting rapid systemic stress signaling [234].

Compared to electric shock or conspecific alarm substance models, restraint stress offers an intermediate level of ethological validity and engages both psychological and physical domains of stress. However, unlike validated multi-stressor paradigms (e.g., 14-day CUS/UCS), it lacks the chronic unpredictability required to model long-term PTSD features such as sustained hyperarousal or emotional numbing.

On the other hand, given its controlled nature, immobilization stress in zebrafish provides a powerful approach to studying short-term neurochemical and behavioral responses to aversive experience. Its predictable structure makes it ideal for studying anticipatory anxiety, while unpredictable variants may better approximate hypervigilance-like states. Future applications of this model should focus on systematic comparison of chronic vs. acute immobilization, as well as integration with pharmacological interventions targeting oxidative stress and neurotransmitter imbalance [66]. Until then, immobilization paradigms are best used to complement—not replace—validated chronic multi-stressor models such as CUS/UCS.

#### 5.2.2. Immobilization Stress and PTSD

Immobilization stress triggers a cascade of physiological and behavioral responses in zebrafish that closely parallel acute stress reactions in other species. It makes it a valuable model for understanding discrete PTSD-related phenotypes, particularly those linked to helplessness, lack of control, and anticipatory anxiety. The acute stress from immobilization activates the HPI axis, leading to a significant increase in cortisol release, a hallmark of the stress response also observed in mammalian models. The behavioral consequences of immobilization stress are highly indicative of an anxiety-like state, as evidenced by increased bottom-dwelling, reduced exploratory behavior, and heightened thigmotaxis (edge-clinging behavior) which are measured in the novel tank diving test and light–dark preference test.

Immobilization stress induces oxidative stress in zebrafish brains, as indicated by elevated lipid peroxidation and altered antioxidant enzyme activity, particularly a reduction in catalase activity [231]. These findings suggest that oxidative damage plays a critical role in the neurobiological effects of acute stress, which is relevant to PTSD pathology [116,117,235,236]. Additionally, zebrafish subjected to acute restraint stress exhibit GABAergic dysfunction, with studies reporting significantly diminished GABA levels in the brain, reinforcing the role of GABAergic inhibition in stress adaptation and PTSD-related symptoms [233,237]. A recent study further established a direct link between restraint stress and depressed GABAergic transmission: restrained zebrafish displayed reduced GABA release and downregulated c-fos expression in the telencephalon, a key brain region involved in fear processing and emotional regulation [238].

In addition, immobilization stress appears uniquely suited to model psychological trauma dimensions such as uncontrollability and behavioral passivity—central constructs in PTSD etiology. Acute spatial restriction-based stress has been associated with metabolic alterations, including shifts in purinergic signaling and ATP hydrolysis, highlighting broader systemic effects of stress on neurochemical regulation. Nevertheless, as our systematic review indicates, immobilization paradigms are underutilized in chronic protocols and lack the multi-dimensional stress exposure needed to model full PTSD syndromes. Their current utility is best framed as complementary to multi-stressor paradigms such as CUS/UCS, rather than standalone substitutes.

Taken together, these findings support immobilization stress as a targeted model for investigating the acute physiological and molecular mechanisms underlying trauma-induced anxiety and helplessness (Table 5). Future studies should explore chronic restraint designs, sex-specific vulnerability patterns, and multi-modal assessments to improve the paradigm’s translational reach. Combining immobilization with validated chronic stress protocols (e.g., 14-day CUS/UCS) may also help model more complex PTSD phenotypes involving sustained dysregulation and comorbid behavioral profiles.

### 5.3. Confinement Stress Models

#### 5.3.1. Application of Confinement Stress

Confinement stress involves placing zebrafish in small containers or restricting their swimming area to induce stress. This method capitalizes on zebrafish’s natural preference for open spaces and shoaling behavior. Due to its chronic and predictable nature, this paradigm is particularly suited for exploring long-term effects of social restriction, environmental monotony, and resource limitations which are often implicated in PTSD pathogenesis.

Importantly, confinement is generally considered a mild-intensity stressor, and the outcomes should be interpreted accordingly. It provides a useful model for studying social withdrawal, diminished social motivation, and altered group dynamics, especially in the context of subthreshold or cumulative stress exposure. For example, Wong et al. (2010) confined zebrafish individually in small beakers for 1 h to induce stress [239]. The confined zebrafish exhibited elevated cortisol levels and changes in social behavior upon return to the group, such as reduced aggression and altered dominance hierarchies.

#### 5.3.2. Confinement Stress and PTSD

Confinement stress leads to acute activation of the HPI axis, increasing cortisol secretion and modifying behavior [240]. The alterations in social interactions following confinement are relevant to PTSD, where social avoidance and interpersonal dysfunction are common symptom domains [16,241]. Research by Pavlidis et al. (2015) showed that confinement stress resulted in significant changes in brain mRNA expression in genes related to stress hormone signaling and immune regulation, including interleukin-related markers and glucocorticoid response genes [32]. These molecular adaptations offer insights into stress–immune crosstalk, a domain increasingly recognized in PTSD neurobiology. However, findings remain inconsistent. One study did not observe stress-induced hyperthermia or increased preference for warmer water following confinement [242], contradicting earlier results by Rey et al. (2015) [243], who found that pre-disturbed fish showed enhanced warm-water preference, possibly reflecting a shift in thermal comfort-seeking under stress. Compared to validated chronic multi-stressor paradigms such as 14-day CUS, confinement stress models seem to be more limited in both scope and intensity. They do not capture unpredictability or fear-based responses and primarily elicit subclinical physiological and social disruption. As such, they are best used to model mild chronic stress, particularly where ethical considerations or protocol simplicity are prioritized. Nonetheless, their strength (Table 6) lies in the ability to measure reintegration dynamics and social cohesion recovery after stressor exposure, offering a narrow but useful window into PTSD-relevant phenomena such as relational withdrawal and social rank instability.

### 5.4. Exposure to Acute and Prolonged Stressors

#### 5.4.1. Application of Stressors

Exposure to acute and prolonged stressors in zebrafish allows for the study of both short-term, high-intensity stress events and extended, cumulative stress exposures. Importantly, the flexibility of these paradigms—ranging from seconds to weeks—permits manipulation of both duration and intensity, enabling researchers to model a spectrum of stressor profiles.

**Figure 4:**
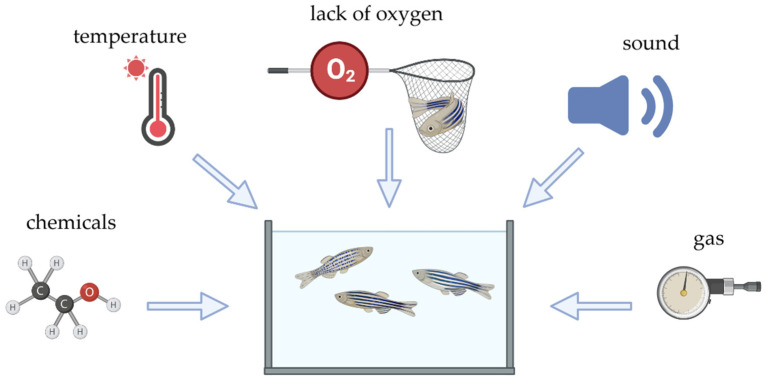
Different stressors that can be applied either acutely or in prolonged manner: chemical substances, temperature, lack of oxygen, sound, and gases in the water (other stressors can be used as well).

Zebrafish can be exposed to various acute stressors, such as temperature changes (e.g., rapid or sustained shifts), air exposure by briefly removing zebrafish from water, or chemical stressors such as caffeine, ethanol, or conspecific alarm substances (CAS) (Figure 4). Abrupt environmental stressors simulate single-event traumas, while chronic exposures (e.g., prolonged thermal shifts) better mimic resource depletion and persistent physiological strain.

Cachat et al. (2011) [144] studied the effects of acute stress induced by net handling and air exposure. The zebrafish exhibited immediate increases in cortisol levels and anxiety-like behaviors, such as increased bottom-dwelling and reduced exploration. Abrupt changes in lighting are also used as acute stressors, particularly in larval assays examining thigmotaxis [244]. Various psychoactive substances modulate anxiety-like behavior and help stratify zebrafish into resilient and vulnerable subtypes. This has been used in combination with light–dark tests to assess stress reactivity and neurochemical correlates [245,246]. On the other hand, the outcome of acute stress by a certain factor may depend on other factors. For instance, the acute cold stress of the same extent and duration was more tolerant in zebrafish fed by specific diet counterbalancing oxidative stress and, in turn, was much less tolerant under high oxidative conditions [247]. Similarly, acute stressing in zebrafish by net handling with air exposure can be relieved under pre-stress administration of melatonin which is known to decrease cortisol production and oxidative stress [64].

Beyond acute models, middle-term stressors, such as 24 h white noise exposure, induce anxiety-like behaviors in both young and adult zebrafish, including enhanced bottom-dwelling and shoaling [88,140,248]. Interestingly, stressed individuals also exhibited increased social attraction, suggesting crowding may serve as a buffering mechanism—though this contradicts findings that stress signals can be chemically transmitted to conspecifics, triggering social avoidance [18].

Prolonged stressing is used to induce sustained changes in the neurophysiological and behavioral features. Chronic unpredictable and strong stress lasting 5 weeks induced a steady increase in whole-body cortisol as well as pro- and anti-inflammatory cytokines (IL-1β, IL-6, IL-10) [249]. In addition, structural changes in telencephalic neurons were observed. Various total durations for the stressing period are utilized, mostly around 4–5 weeks [136] but much longer periods are also reported [250]. Importantly, the duration of chronic stress affects differently on behavior. For example, 5-week chronic stress develops sustained anxiety-like behavior while 12-week stressing with the same stressors produces depression-like behavior. In the first case, both cortisol and norepinephrine levels as well as the expression of pro-inflammatory gene markers in CNS neurons were shown to be elevated. In the latter case, in contrast, the level of cortisol was decreased, serotonin utilization was impaired, and the expression of anti-inflammatory markers was activated [250]. Notably, prolonged thermal stress over 21 days at 34 °C resulted in paradoxical behavior: reduced anxiety-like signs but increased time spent in aversive environments (e.g., upper tank, bright areas) [251]. These outcomes may reflect metabolism-driven behavioral shifts, complicating the interpretation of temperature-based models as direct analogs for trauma.

#### 5.4.2. Acute Stress and PTSD

Acute stress exposure in zebrafish leads to rapid activation of the HPI axis, offering a model for studying early-stage neural, hormonal, and behavioral responses to trauma [252,253]. Common behaviors include thigmotaxis, reduced locomotion, and increased freezing, which resemble core PTSD symptoms such as hyperarousal, risk avoidance, and behavioral inhibition [90,244]. Egan et al. (2009) showed that acute stress alters neurotransmitter dynamics, particularly serotonin and dopamine, both of which are implicated in mood regulation, fear encoding, and PTSD pathology [254]. Also, acute stress is affecting social approach and maintenance in adult zebrafish in a strength-dependent manner [121]. It has been shown that the intensity of stress directly affects the recruitment of cells that reside in the paraventricular nucleus of the hypothalamus and produce corticotropin-releasing hormone [155]. However, if the acute stress is short and not intense enough, its application may result in a lack of stress response. For example, in young zebrafish, the net handling stress for 30 s did not produce any of the specific stress-related changes like alterations in whole-body cortisol level and anxiety-like behavior [255]. These findings clearly demonstrate that the stressor intensity is an ultimately important factor in the overall outcome of stress in zebrafish, similar to numerous findings in humans.

If the chronic stress condition is applied to larval zebrafish, they develop anxiety-like behavior in later life, accompanied by elevated levels of glucocorticoid receptors [256]. Moreover, stress-induced alterations occur after two months post-stressing, indicating their substantially delayed development, likely based on the remodeling of neurophysiological pathways. Relatively short chronic periods of stress (5 weeks) have also been reported [257]. In a very prolonged period of 11 weeks with strong stress, combining various stressors like crowding, vibration, net chasing, social isolation, food deprivation, and many others, the neurophysiological changes in adult zebrafish occur in the form of sustainably high anxiety and elevated level of norepinephrine [258]. On the other hand, many behavioral changes can be alleviated by pharmacological treatment. Therefore, even severe stress does not produce irreversible structural pathological remodeling in stressful situations, at least in the zebrafish stress model.

Despite their utility in probing initial stress responses, acute models lack the chronic unpredictability, emotional salience, and delayed recovery features characteristic of human PTSD. Prolonged but mild stress (e.g., thermal shifts or white noise) can reveal compensatory behaviors but often fall short of modeling persistent dysregulation [259]. These paradigms are best used for mechanistic studies of trauma onset, drug screening, or genotype-specific stress sensitivity, rather than for modeling full PTSD syndromes (Table 7).

## 6. Predator-Based Models in Zebrafish PTSD Research

### 6.1. Behavioral and Hormonal Responses Induced by Predator Exposure

**Figure 5:**
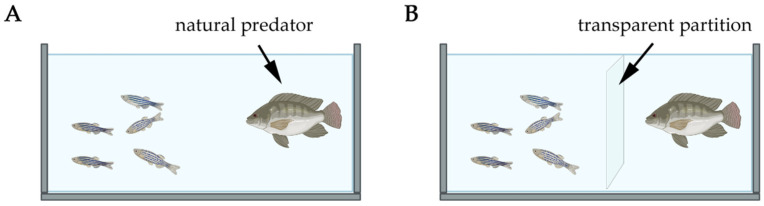
Two distinct types of exposure to predators in zebrafish model of PTSD. (**A**) Direct exposure when each individual fish is at actual risk to be prey. (**B**) Visual exposure via transparent physical partition when fish are not at actual risk of being hunted. Using natural predators for zebrafish (Indian leaf fish) may elicit stronger stress.

Exposure to predators or predator-associated cues elicits robust stress responses in zebrafish (Figure 5), characterized by behavioral and neuroendocrine changes that mirror key features of post-traumatic stress disorder. Repeated or sustained predator exposure leads to increased freezing, reduced exploratory activity, erratic swimming, and startle hyper-reactivity—behaviors analogous to hypervigilance, behavioral inhibition, and arousal dysregulation in human PTSD [4,136]. These behavioral phenotypes are often accompanied by significant elevations in cortisol and alterations in the expression of stress-related genes, such as crf and nr3c1, confirming activation of the HPI axis and its regulatory feedback mechanisms [136,213].

Fish reared in high-predation environments often display adaptive phenotypic plasticity, including greater boldness, faster decision-making, and more efficient shoaling behavior. Paradoxically, they also exhibit lower baseline cortisol levels compared to fish from low-predation environments. This attenuation of neuroendocrine reactivity has been interpreted as a potential marker of chronic stress adaptation or resilience, although these effects are modulated by factors such as age, sex, and prior exposure history [260]. Importantly, such individual variation can obscure straightforward correlations between cortisol levels and behavioral outcomes [260]. This underlines the need for standardized protocols and individual-level tracking in predator-based paradigms.

### 6.2. Influence of Predator Type on Zebrafish Responses

Zebrafish responses to predator cues are strongly shaped by both the type of predator and the modality of exposure. Studies show that zebrafish react more strongly to sympatric predators—those with which they share natural habitats—than to allopatric ones. For instance, exposure to the Indian leaf fish (*Nandus nandus*), a natural predator of zebrafish, results in significantly higher fear-related behaviors than exposure to more visually conspicuous but ecologically unfamiliar predators such as the compressed cichlid [261]. This ecological validity enhances the translational relevance of predator exposure paradigms, especially when the goal is to replicate evolutionary conserved mechanisms of threat detection.

In addition to predator identity, the mode of presentation—whether live, visual-only, or multi-sensory—plays a critical role in shaping stress outcomes. Direct cohabitation with a live predator leads to more pronounced cortisol responses than visual or compartmentalized exposure [139]. However, visual stimuli alone (e.g., through transparent partitions or digital predator animations) are sufficient to induce anxiety-like behaviors and endocrine changes. These findings emphasize the importance of visual threat perception in zebrafish and its utility for modeling PTSD-relevant symptoms such as anticipatory anxiety and contextual fear.

Behavioral markers—including increased time spent at the bottom of the tank, prolonged freezing, and heightened erratic movement—are consistently observed following predator exposure, and these responses scale with perceived threat intensity. Digital predator stimuli, in particular, offer a reproducible and ethically manageable method for inducing stress, while enabling precise temporal control. This approach is especially valuable for high-throughput behavioral screening and neuroimaging applications, where fixed visual stimuli can be paired with real-time monitoring of neuronal activity in larval or immobilized adult zebrafish.

### 6.3. Artificial Predator Models: Looming Dot Stimulus (LDS)

Artificial predator paradigms offer a standardized and ethically sound alternative to live predator exposures. Among these, the looming dot stimulus (LDS) has emerged as a particularly effective model for inducing robust defensive responses in zebrafish. This visual stimulus mimics the expanding shadow of an approaching predator by rapidly increasing the size of a black disk projected on a light background. The LDS paradigm elicits innate freezing, escape swimming, and erratic locomotion—behaviors conserved across vertebrate species including rodents and primates—making it highly suitable for comparative neuroethological studies of fear and threat perception [262,263,264,265,266].

In zebrafish experiments, the LDS is typically delivered via a computer monitor placed beneath or beside a transparent behavioral tank. The parameters of the expanding dot (initial size, expansion speed, contrast, repetition) can be precisely controlled, enabling detailed dose–response analysis. Experimental findings indicate that both the number and intensity of LDS exposures correlate with the magnitude of the stress response, as measured by plasma cortisol levels. For example, Cook et al. (2023) demonstrated that zebrafish exposed to repeated looming stimuli exhibited a cumulative increase in cortisol, confirming the dose-dependent nature of visual threat perception in this model [121].

Importantly, the LDS paradigm allows for non-contact, repeatable threat exposure, eliminating confounds associated with predator variability or physical trauma. This makes it particularly advantageous for dissecting neural circuits involved in threat detection, such as visual pathways projecting to subpallial regions and homologs of the mammalian amygdala and superior colliculus [267,268]. The method is also compatible with in vivo neuroimaging techniques and optogenetic manipulations, enabling direct interrogation of neuronal activity during defensive behavior execution.

In the context of PTSD modeling, the LDS paradigm captures key aspects of acute trauma and visual threat reactivity, offering a platform for high-resolution behavioral phenotyping and neurobiological analysis. Its predictability and control make it ideal for standardized assessments of fear generalization, sensitization, and habituation. Moreover, because it does not require live conspecifics or predators, the LDS paradigm supports ethically optimized research while maintaining high translational potential.

### 6.4. Conspecific Alarm Substance (CAS) as a Predator-Based PTSD Model

Conspecific alarm substance (CAS) is a naturally released chemical signal emitted by zebrafish when their skin is damaged, typically during predation events. This substance serves as an innate warning cue to conspecifics, triggering an acute fear response and facilitating group survival. As such, CAS exposure by adding it directly to the water provides an ethologically relevant, predator-associated stressor for modeling anxiety and post-traumatic behaviors in zebrafish [18,269].

Behaviorally, CAS exposure results in pronounced anxiety-like reactions in zebrafish, including increased bottom-dwelling, erratic swimming, freezing, and avoidance of aversive zones—responses observable in the light/dark preference and novel tank diving tests. Notably, these responses can persist beyond immediate exposure, with heightened anxiety-like behaviors still evident 24 h later [269,270]. This persistence supports the model’s utility in capturing delayed and prolonged stress reactivity, a hallmark of PTSD. However, not all findings confirm robust associative learning. For instance, single CAS exposure did not significantly alter risk assessment in a conditioned place aversion paradigm, even after seven days [269]. Physiologically, CAS exposure elicits significant increases in whole-body cortisol and circulating catecholamines, mirroring the activation of the HPI axis typical of acute trauma responses [18].

Strain-specific and individual differences also modulate the response to CAS. Canzian et al. (2017) demonstrated that both wild-type and leopard strains exhibited increased erratic movements and freezing following CAS exposure [271]. However, only wild-type zebrafish showed enhanced shoal cohesion and reduced inter-individual vertical distance—markers of increased social bonding. These findings point to genetic variation in social stress processing, an area of growing importance in PTSD research. Moreover, population-level variability has been documented: approximately 20% of zebrafish displayed minimal sensitization to CAS, while ~25% exhibited extreme sensitivity, reflecting inter-individual differences in stress vulnerability reminiscent of human PTSD heterogeneity [18].

Nevertheless, concerns remain regarding the associative learning potential of CAS. As in rodent models using predator odors, CAS may function more as a general anxiety cue than a specific trauma-associated signal [266]. Predator odors in rodents have shown inconsistent efficacy in generating robust defensive behaviors (e.g., freezing, escape), often due to their indirect threat valence. Similarly, in zebrafish, CAS appears to trigger non-associative fear responses, limiting its ability to model complex trauma-related learning. This challenge has prompted discussion in the PTSD modeling literature, including recommendations against housing stressed and non-stressed animals together due to potential social transmission of stress cues [272,273,274,275].

Importantly, the coping style of individual zebrafish critically influences CAS response. Animals exhibiting a reactive stress coping style—characterized by passive, cautious behavior—tend to acquire fear memories more rapidly and retain them longer than their proactive counterparts, who are more exploratory and resilient [60]. Reactive fish show stronger freezing and less exploratory behavior upon re-exposure to CAS-associated environments, whereas proactive individuals may require repeated exposures to form equivalent fear associations. These differences underscore the importance of phenotyping stress responses in zebrafish and align with human data on stress sensitivity and PTSD risk.

### 6.5. Relevance and Applications to PTSD Research

Predator-based stress models in zebrafish—including direct predator encounters, artificial stimuli such as the looming dot stimulus (LDS), and conspecific alarm substance (CAS) exposure—constitute a versatile and ethologically relevant platform for investigating PTSD-like phenotypes. These models simulate acute or chronic exposure to threatening stimuli, thereby recapitulating the types of traumatic experiences often implicated in the development of PTSD in humans. Depending on the frequency, duration, and modality of exposure, predator-based paradigms can be applied to model either acute trauma (e.g., single predator encounter or LDS flash) or chronic threat scenarios (e.g., repeated exposure to predator cues), thus allowing researchers to investigate the temporal dynamics of stress responses and their persistence over time.

One of the distinctive advantages of predator models lies in their capacity to engage evolutionarily conserved fear circuits, eliciting robust behavioral changes such as freezing, thigmotaxis, erratic swimming, and shoal cohesion. The behavioral features have a strong parallel to anxiety, hypervigilance, and avoidance behaviors in PTSD. The stress intensity in predator models, although not numerically quantifiable as in electric shock paradigms, can be modulated by using transparent partitions, partial concealment, or restricted escape routes. Similarly, the LDS allows for a fine-tuned adjustment of visual threat intensity and duration. Such flexible design facilitates the experimental alignment with mild, moderate, or high-stressor PTSD paradigms.

Importantly, predator exposure—whether live, artificial, or chemical—activates key neuroendocrine and immune pathways, including the hypothalamic–pituitary–interrenal (HPI) axis, catecholaminergic circuits, and pro-inflammatory signaling. CAS and live predator paradigms have been shown to induce cortisol elevation, modulate gene expression related to neurotransmission and inflammation, and affect social behavior and fear memory retention [4,213]. These biological correlates mirror core features of human PTSD pathophysiology.

Nevertheless, certain limitations warrant attention. Zebrafish do not manifest PTSD as a clinical syndrome, and thus their reactions represent components rather than the full spectrum of PTSD symptomatology. Furthermore, individual variability—driven by genetic background, sex, and coping styles—can introduce heterogeneity into behavioral outcomes, complicating interpretation unless stratified analyses are conducted [60]. Another methodological caveat is the potential lack of associative learning following predator odor or CAS exposure, raising questions about the extent to which these paradigms can simulate conditioned fear and trauma-linked memory formation [266,274].

Despite these challenges, predator exposure models offer a high degree of ecological validity and are technically and economically accessible, enabling high-throughput analysis in both adult and larval zebrafish. Their integration into PTSD research provides a powerful means of exploring the interplay between acute threat processing, chronic anxiety states, and neuroimmune dysregulation. Future directions should prioritize combining predator-based stressors with longitudinal behavioral assays, multi-omics profiling, and real-time imaging to enhance model resolution and mechanistic insight.

In sum, predator-based models—including LDS and CAS—are integral components of the zebrafish PTSD modeling framework, offering diverse experimental formats to dissect the neural, hormonal, and behavioral consequences of trauma. While no single model captures the full complexity of PTSD, their complementary strengths support their continued use as a multi-dimensional platform for pre-clinical PTSD research.

## 7. Complex Stress Models for PTSD in Zebrafish

The use of complex stress models in zebrafish research offers significant advantages for understanding the intricate nature of PTSD. These models allow for the exploration of how different stressors interact to produce the behavioral, physiological, and molecular changes associated with chronic stress. By incorporating gender differences and refining experimental protocols, researchers can gain a deeper understanding of PTSD and develop more targeted therapeutic strategies. Despite the challenges associated with these models, including the variability in cortisol responses and the need for careful experimental design, they represent a valuable tool in the ongoing effort to unravel the complexities of PTSD.

### 7.1. Chronic Unpredictable Stress (CUS/UCS)

In zebrafish research, complex stress models, particularly those utilizing chronic unpredictable stress (CUS/UCS), have shown significant promise in replicating the multi-faceted nature of real-world stressors. Unlike isolated stress events, real-life stressors are often complex, involving multiple factors such as psychological, physical, and environmental elements, similar to the stress experienced by individuals with PTSD, particularly in combat scenarios [6]. The CUS/UCS model in zebrafish attempts to capture this complexity by subjecting the fish to a series of unpredictable stressors over time, thereby closely mimicking the chronic stress conditions that humans often endure [136]. This approach has been effective in inducing changes in anxiety-related behaviors and altering neurotransmitter levels, thereby providing a closer approximation of the chronic stress experienced by humans [4,15].

Various interpretations of CUS/UCS protocols have been described in the literature, each with its own set of stressors and methodological nuances. One approach involved exposing zebrafish to a wide range of stressors twice daily for either 7 or 14 days, following a two-week habituation period [113]. Another approach used five different stressors ranging from net chasing to water pH change applied to zebrafish larvae (starting from 10 dpf) intermittently, twice a day using two stressors per day, for a week [15]. Some protocols may use a substantially prolonged period of various unpredictable stressors, e.g., up to 4 weeks in one study [136] and up to 12 weeks in another study [250]. The stressors may include restraint stress, heating and cooling of tank water, social isolation, crowding, exposure to a predator, low water levels, repeated tank water replacement, sudden water pH change, and net chasing. This intensive use of multiple stressors targets different aspects of the zebrafish’s physical and psychological comfort. The frequent exposure to varied stressors over an extended period closely mimics the persistent and multi-faceted nature of chronic stress in humans. Zebrafish subjected to this regimen showed reduced locomotor activity, increased pigmentation, and significantly elevated cortisol levels, indicative of a robust stress response.

Another interpretation described in the literature involves exposing zebrafish to seven different stressors in a randomized order over seven days, with two stressors applied each day at random times between 9 a.m. and 4 p.m. [169]. The stressors included rapid tank changes, abrupt temperature shifts (both cooling and heating), lowered water levels, net chasing, crowding, and social isolation. The randomization of stressor order and the timing of their application add a layer of unpredictability, preventing the fish from anticipating the stressor and more closely mirroring the unpredictability of real-life stressors. After seven days of exposure, zebrafish exhibited increased anxiety-like behaviors in the novel tank test and a reduction in basal cortisol levels, suggesting that while the protocol effectively induces stress-related changes in behavior, there may be an adaptation to the stressors over time.

A third interpretation of the CUS/UCS model integrates predator exposure with other traditional CUS/UCS stressors [104]. In this variation, zebrafish were subjected to a combination of stressors, including the heating and cooling of tank water, predator exposure, crowding, low water levels, tank changes, and net chasing. Predator exposure introduces a significant psychological stressor that directly mimics a life-threatening scenario. This approach led to heightened anxiety-like behaviors, elevated cortisol and reactive oxygen species (ROS) levels, and disrupted social behavior, making it particularly relevant for PTSD models.

Gender differences significantly influence the response of zebrafish to CUS/UCS protocols. Research indicates that male zebrafish tend to exhibit increased aggression following exposure to chronic stress, a reaction not typically observed in females [97]. Interestingly, at baseline, females displayed more aggressive behavior compared to control males. Chronic stress also led to increased locomotor activity in females, which was not observed in either stressed or control males, highlighting a gender-specific response to stress. Additionally, stressed males generally showed elevated cortisol levels compared to other groups, whereas cortisol levels in females remained consistent regardless of stress exposure [97].

On a molecular level, CUS/UCS led to elevated levels of cytokines and markers of neuroinflammation, with increased expression of genes associated with stress, inflammation, and cell death in both males and females. However, the anti-inflammatory cytokine IL-4 did not show a significant difference in expression between stressed and control groups, highlighting the complexity of the inflammatory response to chronic stress [6]. Despite these findings, it is also noted that the elevated cortisol levels observed in zebrafish subjected to CUS/UCS may contribute to the disruption of social structures within groups, reflecting a loss of resilience and behaviors similar to those seen in depressive-like states [113].

Several factors may account for the observed differences in cortisol responses among various CUS/UCS protocols and between genders. The timing of the stressor application plays a crucial role, with stressors applied during the resting phase (night-time) having a more pronounced effect on cortisol levels than those applied during the active phase (daytime) [276]. Variations in experimental protocols, including differences in the types, duration, and timing of stressors, also likely contribute to the diverse cortisol responses observed across studies. For instance, longer exposure periods and different combinations of stressors might result in distinct physiological stress outcomes [113,169]. Handling and environmental factors, such as the specific procedures used to manipulate the zebrafish and the conditions within their tanks, can also influence cortisol levels differently depending on the experimental setup. Additionally, biological variability and sex can introduce differences in cortisol responses, with males generally exhibiting higher cortisol levels following chronic stress compared to females [97]. The dynamics of the cortisol stress response (duration, intensity) also play a significant role, as zebrafish typically exhibit a rapid and sustained increase in cortisol following acute stress, with levels returning to baseline approximately two hours after the stressor is removed [4,32].

Recent evidence from a systematic review confirms that 14–15 day CUS/UCS protocols represent the optimal duration for inducing PTSD-like phenotypes in zebrafish [19]. These paradigms consistently produce severe and persistent anxiety-like behaviors, sustained hypercortisolemia, and upregulation of pro-inflammatory genes (e.g., TNF-α, IL-6), closely resembling mammalian PTSD signatures. Conversely, shorter (7-day) protocols are associated with milder behavioral effects and transient neuroendocrine responses, while extended protocols (≥21 days) show diminished responsiveness presumably due to physiological adaptation or exhaustion (Table 8). On the molecular level, 14-day protocols were shown to induce GR/NR3C1 downregulation, CRH overexpression, and oxidative stress marked by elevated 8-OHdG and reduced SOD/CAT activity. These changes closely mirror those seen in human PTSD cohorts. Furthermore, sex-specific patterns highlighted in the review suggest that males respond with sharper cortisol spikes, while females exhibit persistent downregulation of BDNF and impaired GABAergic signaling, even in the absence of cortisol elevation. To enhance reproducibility and translational value, CUS/UCS paradigms should standardize stressor intensity and timing. The most effective protocols utilize 2–3 daily stressors over 14–15 days, randomized in type and timing, and delivered during the inactive circadian window (09:00–16:00). Recommended outcomes include novel tank test (freezing time), cortisol ELISA, and qPCR for nr3c1, crh-a/b, il6. Cohorts with <20% mortality should be excluded to minimize non-responder bias [19].

### 7.2. Time-Dependent Sensitization (TDS) as a Distinct Method or Phenomenon

Time-dependent sensitization (TDS) is a phenomenon observed in zebrafish and other species that reflects the progressive amplification of behavioral and physiological responses following repeated exposure to a stressor. With each successive re-exposure, the organism’s response becomes more intense and enduring, even in the absence of escalating stimulus intensity. This process parallels the sensitization of stress circuits in human PTSD, where individuals exhibit heightened responses to previously neutral or mildly aversive cues after trauma exposure [4,18].

In zebrafish, TDS has been leveraged as a stress–restress paradigm, mimicking the real-world scenario where an initial trauma is followed by repeated triggers or reminders of the traumatic experience. It makes persistent neurobehavioral alterations, including increased locomotor activity, prolonged freezing, disrupted shoaling behavior, and heightened cortisol secretion, resembling core features of PTSD such as hyperarousal, re-experiencing, and stress sensitization [18]. From a translational perspective, the TDS model captures the delayed and cumulative nature of trauma progression. For instance, predator odor exposure in rodents leads to persistent hyperarousal only after an incubation period, suggesting neuroplastic changes that unfold over time—a phenomenon mirrored in zebrafish subjected to repeated exposure to CAS. In these fish, anxiety-like behaviors remain elevated 24–48 h post exposure, with some individuals demonstrating extreme behavioral sensitization and others showing minimal response [18,277].

At the neurobiological level, TDS is associated with long-lasting changes in cortisol dynamics, monoaminergic signaling, and neuroinflammation [278]. Repeated CAS or predator exposure not only alters behavior but also increases expression of stress axis genes and markers of neuroimmune activation. This model allows researchers to probe the temporal limits of stress resilience, testing how neuroendocrine and molecular reserves buffer against the accumulation of trauma-induced damage [279].

Importantly, the TDS paradigm facilitates the experimental dissection of PTSD heterogeneity, offering a scalable and ethologically valid framework to study individual susceptibility and delayed trauma emergence. Its utility lies in bridging the gap between acute and chronic models, highlighting how repetition and the memory of trauma—not just intensity—shape long-term outcomes.

## 8. Early Life Interventions

Early developmental periods are highly sensitive windows during which environmental and physiological inputs can permanently shape neurobiological function and behavioral phenotypes. In zebrafish, the first post-fertilization days (dpf) represent a critical stage characterized by rapid morphogenesis, dependence on yolk reserves, and immature stress regulatory systems. This high plasticity renders zebrafish embryos and larvae particularly susceptible to perturbations, making them a powerful model for studying the long-term consequences of early life stress and its relationship to PTSD-like states.

### 8.1. Environmental and Mechanical Stressors

Exposure to physical or environmental stressors during the early stages of zebrafish development induces a wide range of structural and behavioral alterations. For example, daily administration of heterogeneous stressors from 1 to 6 dpf, such as temperature shifts and mechanical agitation, was shown to increase immobility and induce craniofacial malformations, including jaw shortening and a wider cranium [280]. These morphological defects were accompanied by molecular changes, including miR-29a overexpression and downregulation of collagen genes, indicating that early life stress disrupts transcriptional control of developmental programs.

Light exposure is another potent environmental factor influencing early neurodevelopment. Larvae raised in constant darkness fail to establish normal circadian locomotor rhythms and display decreased activity levels and reduced survival [55]. Transitioning to light–dark cycles at 5 or 10 dpf significantly rescues these deficits, illustrating the role of photoperiodic entrainment in early-life neurobehavioral organization [55,61].

### 8.2. Pharmacological Stressors and Epigenetic Programming

Pharmacological manipulation during early development has also been instrumental in elucidating stress axis programming. Notably, prednisolone exposure in zebrafish embryos has been shown to induce site-specific methylation of the nr3c1 promoter (CpGI2), leading to reduced glucocorticoid receptor (GR) expression and long-term dysregulation of the HPI axis [4,179]. This mimics similar patterns observed in human cohorts exposed to early adversity, where NR3C1 methylation is linked to PTSD vulnerability, HPA axis hyperactivity, and altered CRH dynamics [158,281,282]. Behaviorally, adult zebrafish that experienced early glucocorticoid exposure exhibit elevated basal cortisol levels, reduced stress resilience, and alterations in neurotransmitter gene expression, mirroring chronic PTSD-like profiles [179]. Importantly, the increasing of cortisol in culture medium resulted in different effects in AB and TL strains in 5-day zebrafish larvae, supporting distinct activity of HPI axis in the two strains [283]. This evidence highlights that careful selection of zebrafish strains and subsequent interpretations of the obtained data should be always warranted.

### 8.3. Considerations in Modeling and Paradigm Classification

Unlike adult models, early life paradigms cannot be easily classified into chronic vs. acute or intermittent, as well as into predictable vs. unpredictable categories. This is because early zebrafish are still undergoing neurodevelopmental maturation and lack fully functional endocrine and immune systems. Note that mild vs. strong or single-stressor vs. multi-stressor paradigms may comply in early-life zebrafish models as they do not rely on durable periods sensitive to the organism’s development but simply reflect the overall intensity of stress. As such, the timing of stressor applications relative to developmental milestones is more critical than the nominal strength or duration of the stressor itself.

Moreover, inter-individual variability in developmental progression—even within synchronized clutches—adds an additional layer of complexity. Differences in stress sensitivity and developmental stage can profoundly influence experimental outcomes, especially when using molecular endpoints or behavioral assays that rely on mature neuroendocrine function. In this context, life stage itself becomes a key experimental variable, rather than a fixed background feature. Researchers must carefully consider developmental timing, window of exposure, and recovery periods when designing early life intervention studies.

## 9. Distinct Features of Zebrafish Compared to Rodents

Zebrafish exhibit several unique features and behaviors that distinguish them from traditional rodent models, making them particularly valuable for specific types of research.

### 9.1. Color

One of the distinct characteristics of zebrafish is their ability to change color in response to various stimuli. When zebrafish experience fear, such as during freezing or erratic movements, they often become pale, especially against a light background. Conversely, when they are excited or aggressive, their color becomes more vibrant, displaying chatoyant hues with pronounced dark-blue stripes. This color change can be visually rated and compared to control groups using a scoring system: 1—for pale, 2—for lighter than normal, 3—for normal, 4—for darker than normal but not chatoyant, and 5—for fully chatoyant with dark-blue stripes [113].

### 9.2. Shoal Cohesion

Shoaling behavior, or the tendency of zebrafish to swim in groups, is another distinct feature that sets them apart from rodent models. Zebrafish naturally prefer to swim in cohesive groups, a behavior believed to be an evolutionary strategy to protect against predators [284]. Unlike rodent studies, which often use solitary animals, zebrafish experiments can incorporate multiple fish in a test tank to maintain natural shoaling behavior. Shoal cohesion is measured against a control group using a scale: 1—for complete lack of group cohesion or interaction, 2—for loose or partial shoaling, 3—for normal shoaling behavior, and 4—for increased shoal cohesion [113,285].

The translational relevance of shoaling behavior in zebrafish lies in its potential link to traits like behavioral inhibition and neuroticism, which are pertinent to internalizing disorders such as phobias, generalized anxiety, and depression. It has been found that the average distance between individual animals shoaled together is shorter in adult zebrafish stressed by noise [88]. Interestingly, studies have shown that shoal cohesion decreases following treatment with anxiolytics like benzodiazepines or ethanol, paralleling the effects seen in reducing anxiety-related behaviors in humans [285]. Additionally, acute cold stress, such as exposure to low temperatures (18 °C and 10 °C) for 48 h, has been found to increase shoaling behavior [57]. This adaptation is thought to be a strategy for conserving energy while foraging and escaping predators under cold stress conditions.

These features—color changes and shoal cohesion—illustrate the unique behavioral and physiological responses of zebrafish, providing valuable insights that are not easily replicated in rodent models. Understanding these distinct parameters is crucial for leveraging zebrafish in studies related to stress, anxiety, and other neuropsychiatric conditions.

### 9.3. Neural Injury Response in Zebrafish and Its Implications for PTSD Modeling

Zebrafish have an innate ability to recover from severe spinal cord injuries, which contrasts sharply with the limited regenerative capacity observed in rodents and humans, including rodents. This distinction is primarily due to the highly plastic and regenerative nature of the zebrafish’s CNS. In zebrafish, severe spinal cord injury triggers a complex regenerative process involving various cell types, including neurons, glial cells, and progenitor cells, all working in concert to restore function [286].

Following spinal cord injury, zebrafish exhibit robust neurogenesis, particularly of glutamatergic and GABAergic neurons, which helps re-establish the excitatory/inhibitory balance disrupted by the injury. This process involves not only the proliferation of new neurons but also the plasticity of existing neurons, which adapt to the injury by acquiring regenerative gene expression profiles. For example, injury-responsive neurons (iNeurons) are identified as critical players in this regenerative process, showing a neuroblast-like gene expression signature that is essential for functional recovery [286]. This ability to regenerate neurons and regain function contrasts with the situation in mammals, where spinal cord injuries typically lead to permanent functional deficits due to the formation of inhibitory scar tissue.

The implications of these findings for PTSD modeling in zebrafish are significant. In rodent models of PTSD, traumatic neural injuries often result in permanent damage, making the effects of trauma and stress persistent and more challenging to study over time. However, in zebrafish, the innate ability to recover from severe neural injuries might lead to outcomes that do not fully translate to mammals, particularly when modeling chronic or irreversible damage seen in human PTSD. This difference underscores the importance of careful consideration when using zebrafish as a model for PTSD, especially in studies involving traumatic neural injuries.

## 10. Limitations of Zebrafish Models in PTSD Research

Zebrafish offer significant advantages as a model organism for PTSD research, including genetic tractability, rapid reproduction, and conserved neuroendocrine pathways. However, there are critical limitations that must be considered when translating findings from zebrafish to mammalian and, ultimately, human conditions. Our systematic review, published in 2025, identified several major constraints in the zebrafish PTSD literature [19], which are summarized and expanded upon below (see also Table 8).

First, there are fundamental anatomical and physiological divergences between zebrafish and humans. Zebrafish lack several brain regions that are central to human PTSD pathophysiology, such as the prefrontal cortex [4]. While the zebrafish telencephalon is functionally analogous to the mammalian amygdala and hippocampus, its organizational patterns are distinct and may impact stress response mechanisms. The HPI axis in zebrafish, although functionally similar to the mammalian HPA axis, differs in cortisol kinetics and feedback regulation. For example, zebrafish exhibit more rapid cortisol peaks (15–30 min post-stress) than humans (30–60 min), complicating direct comparisons of stress hormone trajectories [30,287]. The serotonergic system in zebrafish is also distinct, with gene duplications (e.g., two slc6a4a/b serotonin transporter genes) that may alter pharmacological responses compared to mammals [288].

Second, methodological heterogeneity is a major concern. There is considerable variability in stress induction protocols (e.g., CUS/UCS duration, predator exposure methods), behavioral assays, and outcome measures, which limits reproducibility and comparability across studies. Our systematic review found that only a minority of studies reported power calculations, and nearly a third exhibited inadequate control group quality. Environmental sensitivity—such as sensitivity to water temperature, light cycles, water salinity and pH, oxygen levels, tank background, etc.—introduces additional confounding variability that is less pronounced in rodent models [2]. Standardization of methodologies and adherence to consensus guidelines for reporting animal research, for example, “Animal Research: Reporting of In Vivo Experiments (ARRIVE 2.0)” [289], are crucial for improving the reliability and translational value of zebrafish PTSD models.

A third gap is the insufficient exploration of sex differences. Few studies have compared male and female zebrafish in contrast to well-documented sex differences in PTSD prevalence and symptomatology in clinical populations. This limitation is significant given the growing evidence that stress responses differ between sexes in both humans and zebrafish. Future research should incorporate sex-based analyses to improve the translational relevance of zebrafish PTSD models.

Technical constraints further limit the utility of zebrafish for PTSD research. The small size of zebrafish precludes longitudinal blood sampling for cortisol measurement without euthanasia, hindering time-course studies of stress hormone dynamics. The relatively small brain size also restricts region-specific analyses of neurochemical and molecular changes.

Cognitively and emotionally, zebrafish cannot replicate the complex psychological symptoms of human PTSD, such as intrusive memories, nightmares, or guilt. While zebrafish exhibit anxiety-like behaviors and social withdrawal, these do not fully capture the multi-faceted nature of human PTSD. This fundamental limitation necessitates caution when extrapolating findings from zebrafish to human pathophysiology and treatment.

Pharmacological considerations also present challenges. Drug metabolism and pharmacokinetics differ substantially between fish and humans, and the aquatic environment introduces unique challenges for drug administration and dosing. For example, fluoxetine requires much higher concentrations in zebrafish than the therapeutic plasma levels used in humans [290]. The effects of drug combinations and long-term treatments remain incompletely characterized in zebrafish PTSD models.

Additionally, the regenerative capacity of zebrafish, particularly their ability to recover from severe neural injuries, presents both an advantage and a limitation. While this capacity allows for the study of neuroregeneration and recovery, it also means that zebrafish may not accurately model the irreversible damage and chronic neural deficits associated with PTSD in humans [286,291].

In summary, zebrafish provide a valuable model for studying certain aspects of PTSD, but significant limitations must be considered (Table 9). These include anatomical and physiological differences, methodological variability, lack of sex-specific analyses, technical constraints, limited cognitive and emotional complexity, pharmacological discordance, and unique regenerative capacities. Recognizing these limitations is crucial for accurately interpreting research findings and developing effective therapeutic strategies. Future research should prioritize standardized protocols, sex-specific designs, and multi-model validation to bridge translational gaps and enhance the applicability of zebrafish PTSD models to human conditions.

## 11. Conclusions

Zebrafish offer a robust and powerful model for studying the mechanisms underlying PTSD and other stress-related disorders in humans, providing valuable insights into both immediate and long-term effects of stress at behavioral, molecular, and physiological levels. One of the significant strengths of zebrafish as a model organism is their ability to mirror the stress response through the HPI axis, analogous to the HPA axis in mammals [13,193,293,294,295]. Another great advantage is that zebrafish embryos and larvae are fairly transparent when using them in direct and real-time observations of neural activity, developmental changes, and various (intra)cellular processes like Ca^2+^ transients [296,297], offering a distinct advantage in studying stress responses during early life stages. It has also been demonstrated recently that live adult zebrafish (while restricted and intubated) can be used for long-term (day-long) fluorescent measurements too, extending the usability of the zebrafish model for many studies including cancer development and tissue regeneration [298,299]. The latest developments in the optical methods and techniques further facilitate achieving the precise and long-term in vivo imaging of neuronal activity in small-bodied organisms like intact (adult or young) zebrafish [300].

Notably, research made in the last several years indicates that zebrafish, and likely other fish utilized in the experimental studies, possess distinct individual characteristics creating something like “personality”—the concept is discussed in detail in a recent review [301]. This individuality is particularly relevant when exploring stress resilience and susceptibility, as it offers an opportunity to phenotype stress responses more accurately in the context of PTSD. The “personality” of such a simple organism (of course, compared to mammals and humans) makes a great opportunity for those researchers who are focused on stress response phenotyping in the context of individual resilience/susceptibility to stressors.

The rapid development in digital technologies in the last decade provides another very interesting perspective in the precise studying of behavioral and cognitive functions in fish like zebrafish. Traditionally, the movement of an individual fish is tracked by a video recording device, with further analysis of the movement; however, the full procedure takes a lot of time. Currently, the novel approach is one in which a fish is head-fixed and physically immobile but can “move” in a virtual 2D space imitating a Morris water maze [302]. Surprisingly, in this artificial design, the fish is able to learn the maze in a similar way as under natural circumstances and, of great notice, the fish is also able to find a safe place in the virtual 2D arena when tested under a mild electric shock protocol [302]. These innovations provide unique insights into cognitive processing and learning in zebrafish.

In differentiating PTSD-like states in zebrafish from general fear, anxiety, or habituation, our analysis supports a set of core behavioral criteria. Specifically, PTSD-like states in zebrafish are distinguished by persistent avoidance—manifested as at least 7 days of reduced upper-tank exploration in the Novel Tank Test (NTT) [113]; hyperarousal or startle sensitization, reflected in exaggerated darting responses to looming-stimulus presentations with no evidence of habituation across repeated trials [97]; and social withdrawal, evident as a reduction of at least 20% in shoal cohesion that persists for more than three days following exposure to a stressor [101]. Additional hallmarks include failure of habituation, characterized by sustained freezing or thigmotaxis even after repeated exposures to novel environments, and cognitive impairment in the form of contextual avoidance, as indicated by conditioned place avoidance behavior lasting at least seven days [97].

To enhance reproducibility, we recommend standardizing the experimental variable parameters—even though, aside from stress-duration, no systematic data currently link these factors to PTSD-like outcomes. These should therefore be viewed as community-driven best practices until rigorous, parameter-specific studies are available (Table 10). Future studies should implement standardized CUS/UCS protocols with validated behavioral and molecular endpoints, stratify analyses by sex, and adopt multi-modal approaches such as transcriptomics, neuroimaging, and longitudinal hormone profiling to improve reproducibility and clinical relevance.

To summarize, despite the above-mentioned limitations, zebrafish remain a valuable model for PTSD research, particularly for high-throughput screening and genetic studies. Their adaptability to diverse stress paradigms provides an unparalleled opportunity to explore how acute/intermittent/chronic, predictable/unpredictable stressors, as well as their strength, contribute to PTSD mechanisms. Through continued innovation, zebrafish have the potential to contribute significantly to our understanding of PTSD and the development of novel therapeutic strategies.

## Figures and Tables

**Table 1:** Influence of environmental factors and coping styles on zebrafish stress responses.

Factor	Type of Influence	Outcome	Specific Example	Complexity
Light Cycles	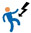 (Environmental Cycle)	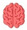 (Circadian rhythm establishment)	Larvae raised in darkness fail to develop normal rhythms [55,56]	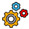
Temperature	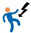 (Environmental Cycle)	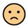 (Stress sensitivity)	Cold stress increases shoaling behavior [57]	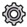
Circadian Rhythms	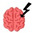 (Biological Rhythms)	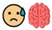 (Behavioral and stress regulation)	Adults maintain strong circadian rhythms under constant conditions [58]	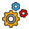
Age-Related Differences	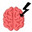 (Biological Aging)	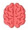 (Stress response changes)	Older zebrafish show reduced locomotor activity and different cortisol responses [2]	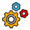
Stress Coping Styles	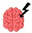 (Behavioral Trait)	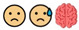 (Stress response, learning, and memory)	Reactive zebrafish exhibit higher cortisol levels, more rapid learning, and longer retention of fear memories, compared to proactive individuals [59,60]	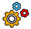

Visual Symbols: Physical Stressor: 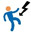; Non-Physical Stressor: 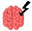; Cognitive or Behavioral Regulation: 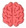; Fear: 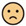; Anxiety: 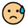; Simple Influence: 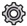; Complex Influence: 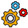.

**Table 2:** Sex-related differences in stress responses in zebrafish.

Aspect/Endpoint	Male Zebrafish	Female Zebrafish	References
Basal Anxiety-Like Behavior	Lower basal anxiety; greater exploration of upper tank; less freezing; lower plasma/brain estradiol and cortisol	Higher basal anxiety; more bottom dwelling, more freezing and immobility; higher estradiol and cortisol	[93,94]
Acute Stress-Induced Cortisol	Higher stress-induced cortisol surge	Lower stress-induced cortisol; enriched or group housing blunts any sex differences	[95,96]
Chronic/Unpredictable Stress (UCS)	UCS increases aggression and cortisol	UCS does not change aggression or cortisol; females more resilient/adapted	[97]
Social and Environmental Modulation	Group housing, enrichment, fluoxetine/diazepam lower cortisol and anxiety—but blunted in enriched/isolated tanks; males show greater modulation	Similar blunted stress response with enrichment/pharmacological agents	[95,96]
HPI Axis Gene Expression	Lower crha, crhr2, actha; higher crhbp (lower HPI reactivity)	Higher crha, crhr2, actha; lower crhbp (higher HPI reactivity)	[93]
Neurotransmitter Profiles (5HT, DA)	Higher brain serotonin and dopamine	Lower serotonin and dopamine	[93]
Aggression (Baseline and UCS Effect)	Lower baseline aggression; UCS increases aggression	Higher baseline aggression; UCS does not further increase aggression	[97]
Transcriptomic and Plasticity Response	Bold lines: strong transcriptomic reprogramming after social defeat; moderate behavioral flexibility	Shy lines: pronounced behavioral flexibility, minimal transcriptomic shifts	[98]
Pain/Nociception	No significant sex difference	Similarly to males; modulatory effects are environmentally mediated	[99]

**Table 3:** Summary for various models for studying PTSD in zebrafish.

Model	Type of Stressor	Outcome	Stressor	Ethological Validity	Etiological Validity	Model Complexity	Stress Paradigm (Duration, Predictability, Strength)
Predator Exposure Models	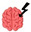	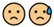	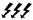	●●●	●●●	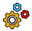	Chronic, Predictable or Unpredictable, Strong
Conspecific Alarm Substance (CAS)	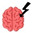	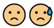	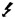	●●	●●	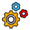	Acute (Intermittent), Unpredictable, Mild
Chronic Unpredictable Stress (CUS/UCS)	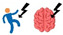	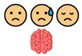	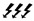	●●	●●●	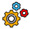	Chronic, Unpredictable, Mild or Strong
Looming Dot Stimulus (LDS)	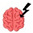	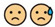	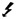	●	●	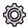	Acute (Intermittent), Predictable or Unpredictable, Mild
Social Isolation Models	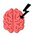	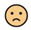	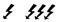	●●	●●	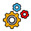	Chronic, Predictable, Mild
Pharmacological Stress Models	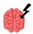	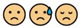	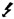	●	●	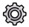	Acute (Intermittent) or Chronic, Predictable, Mild or Strong
Early Life Interventions	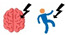	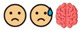	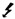	●	●●●	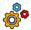	Acute (Intermittent) or Chronic, Predictable or Unpredictable, Mild or Strong

Visual Symbols: Physical Stressor: 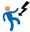; Non-Physical Stressor: 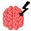; Cognitive or Behavioral Regulation: 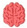; Fear: 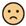; Anxiety: 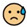; Depression: 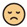; Single Stressor: 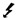; Multiple Stressors: 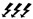; ●●●—High Ethological or Etiological Validity; ●●—Medium Ethological or Etiological Validity; ●—Low Ethological or Etiological Validity; Simple Model: 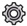; Complex Model: 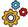.

**Table 4:** Advantages and limitations of electric shock models used for studying PTSD in zebrafish.

**Advantages**	**Description**
Precise, Controllable Stressor	Electric shock intensity, duration, and frequency can be finely calibrated, enabling highly reproducible experiments and systematic manipulation of acute stress parameters.
Robust Behavioral Analysis	Enables detailed study of fear conditioning, contextual learning, avoidance behavior, and punishment sensitivity—core components of PTSD-related cognitive mechanisms. Suitable for high-throughput larval assays (e.g., 96-well setups).
Physiological and Molecular Insights	Activates the HPI axis and induces measurable changes in cortisol and stress-relevant gene expression (e.g., CRF, GR), providing insight into stress hormone dynamics and neuroendocrine activation.
Potential for Ethical Refinement	Under optimized conditions (e.g., brief pulses at ≤3 V), electric shocks can avoid long-term physiological damage, allowing for ethically refined protocols.
Mechanistic Specificity	Ideal for isolating single-event trauma effects, fear memory encoding, and extinction dynamics, especially in genetic or pharmacological manipulation studies.
**Limitations**	**Description**
Ethical and Welfare Concerns	Electrical stimulation can induce distress and is ethically sensitive. Requires justification, strict protocol optimization, and institutional oversight to ensure animal welfare.
Limited Syndromic Validity	Models acute trauma well but fails to reproduce chronicity, unpredictability, and emotional dysregulation of full PTSD. Less suited for modeling, generalized anxiety, or long-term neuroplasticity.
Technical Complexity	Applying shocks underwater demands specialized apparatus, voltage calibration, and shielding to prevent variability—barriers to standardization across labs.
Species-Specific Stress Physiology	Differences in neural conductivity, circuitry, and fear expression may limit direct translation to mammalian PTSD physiology, especially in complex affective domains.
Protocol Inconsistency Across Studies	Variation in shock waveform, duration, frequency, and delivery methods complicates replication and comparative synthesis; emphasizes the need for harmonized, transparent reporting standards.

**Table 5:** Advantages and limitations of immobilization stress models used to study PTSD in zebrafish.

**Advantages**	**Description**
Ethically Acceptable	Immobilization is generally less invasive than predator or electric shock exposure, resulting in fewer welfare concerns.
Relevance to PTSD	Simulates helplessness and uncontrollability—two core psychological dimensions in trauma—thereby enhancing face validity for PTSD-like states.
Physiological and Behavioral Insights	Elicits measurable changes in cortisol, oxidative stress markers, and GABAergic signaling, offering a clear window into acute stress neurobiology.
Simplicity of Setup	Does not require specialized equipment or complex protocols, enabling easier standardization and replication across laboratories.
**Limitations**	**Description**
Potentially Limited Stress Intensity	Stress severity may be lower than in predator or electric shock models, reducing symptom generalizability to severe PTSD phenotypes.
Habituation Effects	Repeated immobilization can lead to rapid desensitization, limiting its use in chronic or long-term stress modeling.
Restricted Scope of Behavioral Outcomes	Primarily models acute anxiety and passivity; does not capture chronic PTSD dimensions like hypervigilance or emotional numbing.

**Table 6:** Advantages and limitations of confinement stress models used to study PTSD in zebrafish.

**Advantages**	**Description**
Simplicity	Easy to implement using basic labware; no specialized equipment needed.
Behavioral Observations	Allows study of social reintegration, altered dominance, and withdrawal, particularly upon return to group housing.
Ethical Considerations	Considered a low-burden stressor, which is advantageous in early screening or chronic low-dose stress protocols.
**Limitations**	**Description**
Stress Intensity	May not reach the threshold needed to induce robust or lasting PTSD-like symptoms.

**Table 7:** Advantages and limitations of acute or prolonged stress models used to study PTSD in zebrafish.

**Advantages**	**Description**
Versatility	A wide range of physical, sensory, chemical, and social stressors can be applied to isolate specific aspects of the stress response.
Stressor Intensity	The intensity of stressor can be varied if necessary, e.g., to study strength-dependent effects.
Rapid Effects	Allows for immediate assessment of stress hormones, behavior, and neural activation following trauma onset.
Accumulated Effects	Allows for long-lasting outcomes of prolonged (chronic) stress, often applied in form of various stressors exchanging each other.
Ethical Ease	Typically low-intensity and non-invasive, making these paradigms suitable for early-stage studies and high-throughput screening.
**Limitations**	**Description**
Transient Responses	Effects may be short-lived and resolve themselves quickly, limiting relevance for chronic PTSD modeling.
Variability	Different stressors and exposure designs may yield inconsistent results across labs and strains.
Limited Scope	Models are not well-suited for studying delayed PTSD symptoms, emotional dysregulation, or fear generalization.

**Table 8:** Comparative efficacy of the duration of CUS/UCS protocols in inducing PTSD-like phenotypes in zebrafish.

Protocol Duration	Anxiety-Like Behavior	Cortisol Dysregulation	Neuroinflammation	Translational Validity
7 days	Moderate	Transient elevation	Mild IL-1β increase	Limited
14–15 days	Severe/Persistent	Sustained hypercortisolemia	TNF-α, IL-6 upregulation	High
21+ days	Variable	Hypocortisolemia	Adaptive tolerance	Low

Note: Translational validity ratings presented here are based on a systematic review [19] and comparative analysis of previously published studies employing various CUS/UCS durations in zebrafish. These ratings synthesize experimental outcomes reported in the literature and do not reflect a new original dataset.

**Table 9:** Major limitations of zebrafish-based models utilized in PTSD research.

Limitation	Type	Impact	Comparison to Mammals	Recommended Solutions
Simpler Nervous System	Physiological	Limits replication of complex PTSD symptoms (e.g., intrusive memories)	Lacks prefrontal cortex; divergent telencephalon organization [4]	Combine with mammalian models for higher-order cognitive validation
HPI vs. HPA Axis Differences	Endocrinological and Genetic	Altered cortisol kinetics and feedback regulation	Faster cortisol peaks (15–30 min vs. 30–60 min in humans) [30]	Use multi-species biomarker panels (CRH, GR, BDNF)
Methodological Variability	Experimental Control	Compromises reproducibility (e.g., CUS/UCS protocols differ across labs)	Less standardized than rodent models [2]	Adopt ARRIVE 2.0 guidelines; share protocol repositories
Sex-Specific Knowledge Gaps	Physiological	Overlooks sex differences in prevalence/mechanisms	Human PTSD shows 2:1 to 3:1 female predominance [292]	Stratify analyses by sex; study estrogen/androgen interactions
Environmental Sensitivity	Environmental	Confounds stress responses (pH, temperature, light)	More dependent on aquatic conditions	Standardize housing
Behavioral Simplification	Behavioral	Anxiety-like behaviors ≠ PTSD psychopathology	No equivalents to nightmares/guilt	Integrate AI-driven ethograms tracking fear generalization
Regenerative Capacity	Neural	May underestimate chronic neural sequelae	Zebrafish CNS regenerates; mammals (including humans) form glial scars [291]	Combine with rodent injury models

**Table 10:** Recommended standardization in research.

Parameter	Recommended Standard	Rationale/Data Support
Tank Size and Shape	NTT: 28 × 15 × 7 cm; OFT: 20 × 20 × 10 cm; LDT: 25 × 15 × 10 cm	No systematic data; proposed to ensure consistent arena geometry.
Lighting Conditions	14 h light (200 lux white LED)/10 h dark, 28 °C water	No systematic data; aligns circadian cues across studies.
Stocking Density	3–5 fish / L	No systematic data; balances social context with minimized crowding.
Water Quality	pH 7.0–7.5; conductivity 300–500 µS/cm; dissolved O_2_ > 6 mg/L	No systematic data; maintains baseline physiological homeostasis.
Stress Exposure Duration	10–15 days; 2 stressors/day; 10–15 min each, random times	Supported by our systematic review as optimal for multi-dimensional PTSD-like phenotypes.
PostStress Testing Windows	24 h, 48 h, 7 days (acute, subacute, persistent)	Common timepoints in CUS/UCS studies; ensures cross-study comparability.

## Data Availability

Not applicable.

## Abbreviations

The following abbreviations are used in this manuscript:

5-HT	Serotonin
ACTH	Adrenocorticotropic Hormone
BDNF	Brain-Derived Neurotrophic Factor
CAS	Conspecific Alarm Substance
CRH	Corticotropin-Releasing Hormone
CUS/UCS	Chronic Unpredictable Test
DA	Dopamine
dpf	days post-fertilization
EE	Environmental Enrichment
GABA	Gamma-Aminobutyric Acid
GC	Glucocorticoid
HPA	Hypothalamic–Pituitary–Adrenal (axis)
HPI	Hypothalamic–Pituitary–Interrenal (axis)
LD	Light–Dark (cycle)
LDS	Looming Dot Stimulus
LDT	Light–Dark Tank Test
NA	Noradrenaline
NPO	Neurosecretory Preoptic (area)
NTT	Novel Tank Test
PTSD	Post-Traumatic Stress Disorder
PVN	Paraventricular Nucleus
TDS	Time-Dependent Sensitization
